# Evolutionary adaptation and mitogenomic diversity of spiders associated with *Nepenthes smilesii* Pitcher Plants in Thailand

**DOI:** 10.1371/journal.pone.0348143

**Published:** 2026-05-04

**Authors:** Fah Lertkulvanich, Natapot Warrit, Mingkwan Nipitwattanaphon

**Affiliations:** 1 Department of Genetics, Faculty of Science, Kasetsart University, Bangkok, Thailand; 2 Center of Excellence in Integrative Insect Ecology, Chulalongkorn University, Bangkok, Thailand; Zoological Survey of India, INDIA

## Abstract

Symbiosis is a close physical interaction between organisms, shaped by species-specific traits and environmental factors. The tropical pitcher plant, *Nepenthes*, exemplifies a predator-prey relationship; however, certain small invertebrates benefit from the pitcher plant without being subjected to predation. For example, spiders from the Thomisidae family inhabit the digestive fluid of the plant without being digested, preying on the organisms captured by the plant. These spiders offer a valuable model for investigating evolution driven by specialized niches compared to free-living relatives. This study characterized the mitogenomes of four spiders residing within the pitchers of *Nepenthes smilesii* in the Phu Kradueng National Park, Thailand: *Thomisus* sp., *Henriksenia* sp., *Epidius* sp. (Thomisidae), and *Pseudopoda* sp. (Sparassidae). The mitochondrial genomes measured 14,731 bp, 15,888 bp, 14,289 bp, and 14,533 bp, respectively, each consisting of 37 genes, characteristic of metazoan mitogenomes. Higher rates of nonsynonymous substitution were observed in the ND2, ND5, and ND6, genes of these pitcher-associated spiders compared to free-living species of the same families, indicating the evolutionary drivers linked to the pitcher plant environment. Distinct gene rearrangements were identified in the three Thomisids, including the duplication of two control region-like sequences in *Henriksenia* sp., while *Pseudopoda* sp. exhibited a typical mitogenome structure. The phylogenetic tree constructed using all 13 protein-coding genes provided significantly stronger bootstrap support compared to the tree based on 600 bp COI sequences. It also revealed that *Thomisus* sp. and *Henriksenia* sp. are clustered within a single monophyletic clade, while *Epidius* sp. was more diverse and formed a paraphyletic group relative to the rest of Thomisidae family. These results provide essential data for systematic studies and illuminate the co-evolutionary genomic signatures of pitcher plant-spider associations.

## Introduction

Spiders are well known for their web-building capabilities and inhabit almost every terrestrial ecosystem on earth. Approximately 50,000 documented species, they represent one of the most diverse groups among arthropods. Spiders act as key predators, regulating populations of various invertebrates and small vertebrates, including small mammals, reptiles, birds, and amphibians in the tropical and subtropical regions. The Araneidae family is particularly notable for constructing orb webs between trees to capture prey. However, most arachnids hunt on the ground instead of building webs, while some live in atypical habitats.

One such group is the pitcher-dwelling spiders [1], which inhabit the pitcher traps of the carnivorous plant *Nepenthes*. These plants have evolved their photosynthetic leaves into pitcher-shaped traps, featuring vivid coloration and nectar to lure a variety of invertebrates and occasionally small vertebrates. Once prey fall into the digestive fluid, *Nepenthes* secretes enzymes to decompose them and absorb essential nutrients. To survive in such an environment, the pitcher-dwelling spiders have developed specialized adaptations that allow them to cling on the inner, wax-coated walls of the pitcher.

Spiders associated with *Nepenthes* predominantly belong to the family Thomisidae (crab spiders). Notable species include: 1) *Henriksenia nepenthicola*, first described by Pocock [1] (originally described as *Misumenops nepenthicla* [2]), which inhabits several host species, including *N. gracilis* and *N. rafflesiana* [3–5]; 2) *Thomisus nepenthiphilus* [6], which shares both its habitat and behavior with *H. nepenthicola*; and 3) *Synema obscuripes* [7], has been documented residing within the pitchers of *N. madagascariensis* in Madagascar.

Additional examples of *Nepenthes*-animal interactions include: 1) Dipteran larvae and tadpoles [8] inhabit the *N. ampullaria* and assist in the decomposition of organic wastes. 2) Hardwicke’s woolly bat (*Kerivoula hardwickii*) in Borneo using the *N. rafflesiana* and *N. hemsleyana* as roosting sites, providing nitrogenous guano as a nutrient source in return [9]. 3) The Summit rat (*Rattus baluensis*) and the Mountain tree shrew (*Tupaia montana*) feed on the sugary exudates of *N. rajah* and *N. lowii* while providing essential nutrients through their excrement [10]. Moreover, a remarkable case of long-term co-evolution is the carpenter ant, *Colobopsis schmitzi* [11], nesting within the hollow tendrils of *N. bicalcarata* and helping to increase prey capture efficiency and maintain pitcher hygiene by removing oversized debris, thereby preventing fluid contamination.

The coexistence between these organisms represents a specialized form of symbiosis. In the case of pitcher-dwelling spiders, it reflects a kleptoparasitism, wherein the spider intercepts prey attracted by the plant’s lures. However, this interaction is partially reciprocal, as the spider’s nitrogenous waste is excreted back into the pitcher, potentially benefiting the plant’s nutrient uptake. This symbiotic dynamic can be modulated by environmental fluctuations [12]. When the prey becomes scarce, pitcher-dwelling spiders may reposition themselves near the pitcher’s lid to increase prey capturing rate. This proactive hunting strategy reduces the prey-escape chance and a common limitation of pitcher’s passive trapping mechanism, thereby influencing the overall nutrient flow within the pitcher ecosystem [13,14].

This study focuses on the spider assemblages associated with *N. smilesii*, a pyrophytic pitcher plant uniquely adapted to seasonal drought and periodic wildfires. This species is indigenous to the pine and savanna forests of Phu Kradueng National Park in Loei, Northeastern Thailand. Although pitcher-dwelling spiders have been primarily documented in regions spanning from Malaysia to Indonesia, which are recognized as hotspots for *Nepenthes* diversity, research within Thailand remains significantly limited. Notably, *N. smilesii* represents a geographically isolated lineage, separated from the other 16 *Nepenthes* species found in the country [15]. This isolation provides a unique ecological context to examine molecular and behavioral co-evolution within a specialized and distinct niche.

Despite their ecological significance, pitcher-plant-associated spiders have undergone limited molecular study. Current data consist mostly of fragmented sequences—such as COI, rRNA, and H3a from *H. nepenthicola* [16]—which presents a significant challenge for comprehensive molecular identification and evolutionary analysis. The mitochondrial genome serves more powerful for resolving recent species divergence and taxonomic ambiguities in highly speciose groups like spiders. Due to its high mutation rate and small effective population size, the mitogenome undergoes more accelerated lineage sorting than the nuclear genome. Furthermore, its high copy number facilitates DNA amplification from preserved or minute invertebrate tissue samples [17–19]. Ultimately, the mitogenome’s compact, contiguous size and conserved structure make it a cornerstone of invertebrate systematics, providing a standardized dataset for comparing diverse spider taxa [18,20,21].

Comprehensive mitogenomic analysis facilitates robust genetic investigations into gene evolution across familial and subfamilial levels, encompassing gene positioning, rearrangements, size variations, codon usage, and transcriptional regulation. Furthermore, it provides high-resolution data for establishing phylogenetic relationships with strong statistical support [22]. To improve our understanding of the evolution of pitcher-associated spiders and related free-living spiders and support future identification of this group, we aimed to sequence the complete mitochondrial genome of the spiders residing within the *N. smilesii* pitchers. These genomic resources will not only elucidate specialized molecular adaptations but also provide a fundamental framework for the future taxonomic identification and systematic study of this enigmatic group.

## Materials and methods

### Specimen collection and behavioral observation

**Fig 1:**
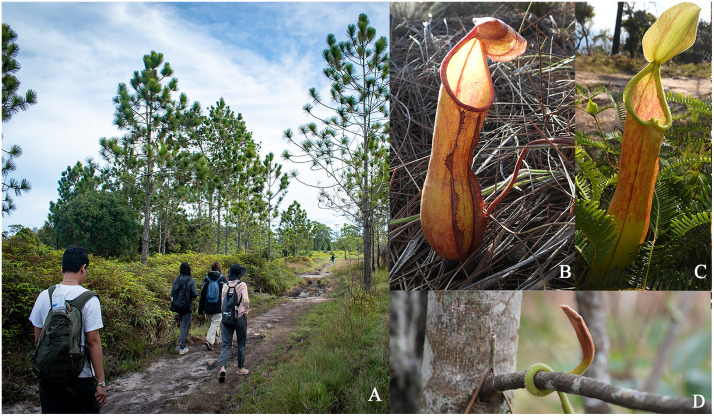
*N. smilesii* found along the natural trails of Phu Kradueng National Park, Thailand. (A) The pine forest and savanna biome. (B) Lower pitcher with an orange freckle pattern. (C) Upper pitcher with a two-color pattern. (D) Tendril development of the upper pitcher to intertwine with objects.

**Fig 2:**
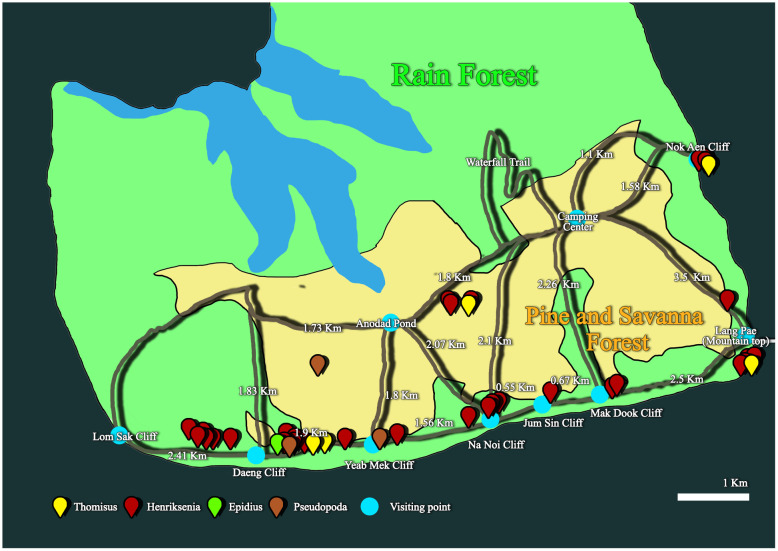
Distribution area of the 40 spider specimens associated with *Nepenthes smilesii* along a natural trail in Phu Kradueng National Park, Thailand. White bar is indicative of 1 km. Pin colors represent specimen collection points.

Field work was conducted at Phu Kradueng National Park, which is located at an altitude of 1,300 meters in the Loei province. Specimens were collected along natural trails near cliff routes characterized by pine forests and savannas, between November 24, 2021, and April 13, 2023 (Figs 1A and Fig 2). Observations and collections were carried out during daylight hours (8.00 to 18.00). Spiders were found residing in both upper and lower pitchers (Figs 1B and 1C), typically in pitchers that did not exceed the height of the supporting shrubbery, regardless of pitcher coloration. A cautious approach was employed to avoid disrupting specimen’s natural behavior. Data regarding the number of individuals per pitcher/plant, microhabitat positioning, and hanging orientation on the inner walls were documented with Nikon P1000 and a Nikon D5600 cameras equipped with a 105 mm macro lens using forceps or vibrations to disturb the spider and observe its response to stimuli. In instances where spiders retreated to the pitcher’s bottom, the lid was carefully excised to allow clear observation and timing of diving and submersion. Following behavioral recording, specimens were extracted from the digestive zone using long forceps, preserved in 95% ethanol, and transported to the laboratory for subsequent molecular and morphological analysis.

### DNA extraction, PCR amplification, and sequencing

Total genomic DNA was extracted from the legs or cephalothorax of the spiders using the FavorPrep™ Tissue Genomic DNA Extraction Mini Kit (Taiwan) The abdomen was kept for further examination of the reproductive organs and was not used in this experiment to prevent microbial contamination. DNA concentration and quality were measured using a Nanodrop spectrophotometer, and samples were stored at a temperature of 4 °C. Partial fragments of DNA barcodes, including COI, 18S rRNA, and H3a genes, were used to identify the species. The COI sequence was amplified to 1,200 bp using a combination of primers LCO and C1-N-2776 [23]. The primer pair for 18S rRNA was 18a2.0 [24] and 18aR [25], while H3a was amplified using primers H3aF and H3aR [26]. PCR reactions were performed in a total volume of 100 µL, consisting of 0.2 mM dNTPs, 1 × reaction buffer, 2 mM MgCl₂, 0.25 U Taq polymerase, 0.5 µM of each primer, and 10–50 ng of spider DNA. Amplification was carried out using an Eppendorf™ PCR Mastercycler Nexus thermal cycler with the following program: initial denaturation at 95 °C for 3 minutes, followed by 40 cycles of 95 °C for 1 minute, 45–47 °C for 1 minute, and 72 °C for 1–1.5 minutes. A final extension was performed at 72 °C for 5 minutes, followed by cooling to 20 °C.

Primers for constructing the overall mitogenome were designed based on the sequences of *Thomisus onustus* and *Ebrechtella tricuspidata* (accession numbers MW832852.1 and KU852748.1, respectively) [27]. These sequences were aligned and compared with other Araneae mitogenomes available in GenBank to identify optimal conserved sites. Six primer pairs were used to amplify overlapping fragments of the complete mitogenome of the pitcher-dwelling spider, with target sizes ranging from 2,000–3,000 bp (Table in S1 Table). The touchdown PCR program was used in this study (Method in S1 Method) to maximize PCR efficiency and specificity [28].

The final PCR products were loaded onto a 1% agarose gel and electrophoresed at 120 V for 30 minutes, followed by visualization under UV light after staining with 0.5 µg/mL ethidium bromide. The target DNA fragments were isolated and purified using the FavorPrep™ Gel/PC Purification Kit and dissolved in 40 µL of elution buffer. The purified PCR products were sent for Sanger sequencing by Macrogen, Inc. (Korea), while larger product sizes were sent for BIT sequencing (Bionics Co., Ltd., Korea).

### Sequence assembly and annotation

Fragments of PCR sequences were checked and assembled using MEGA-11 software (Molecular Evolutionary Genetic Analysis Version 11.0) [29]. MITOS, which is now available as a Galaxy tool (https://usegalaxy.org/), was used to detect the locations of protein-coding genes (PCGs), rRNAs, tRNAs, and the control region, as well as to predict the tRNA secondary structures. The undetected tRNAs were identified by aligning sequences with other spider mitogenomes, locating potential positions, extracting sequences, and resubmitting them to confirm the tRNA sequence. The CGView website (https://cgview.ca/) was used to generate the circular mitogenome map. Gene boundaries were determined by comparing with other spider 83 reference sequences from the NCBI database (Table in S2 Table). Mitogenome composition and relative synonymous codon usage were calculated in MEGA 11.0. Start and stop codons were verified through codon alignment. Tandem repeats in the control region were identified using Tandem Repeats Finder (http://tandem.bu.edu/trf/trf.html). AT and GC-skews were calculated using the formulae (A – T)/(A + T) and (G – C)/(G + C), respectively, to analyze nucleotide composition along the mitogenome. The rate of non-synonymous substitutions (*dN*), rate of synonymous substitutions (*dS*), and the *dN/dS* ratio were analyzed using MEGA-11, to discover the purifying/positive selection of pitcher-associated spiders (Pg020503, Pg305312, Pg071211 and Spa015909) when compared with the free-living spiders (OP650212.1, KM507783.1, KU852748.1, MW832850.1, and MW832852.1) with the same families. The two-sided Wilcoxon Rank-Sum Test with BH post hoc was used to test the differences between the *dN/dS* ratio of pitcher-associated spiders and related free-living spiders [30].

### Phylogenetic analysis

A maximum likelihood analysis was performed to construct two distinct phylogenetic trees. The first phylogeny was based on all protein-coding genes to elucidate the relationships between the study specimens and other arachnids with available mitogenomes. To ensure alignment accuracy, spiders exhibiting atypical gene arrangements were manually reordered to match the mitogenome architecture of the RTA clade, which represents the most diverse group within the Araneae. The other tree was constructed based on 600 bp of partial COI to compare the evolutionary relationships between pitcher-dwelling spiders and closely related taxa for which complete genomes are not available. Phylogenetic reconstruction was performed using the General Time Reversible model with Gamma distribution and Invariant sites (GTR + G + I), identified as the optimal substitution model based on the lowest Bayesian Information Criterion (BIC) and Akaike Information Criterion (AIC) scores. The number of discrete gamma categories was set to five. To account for gaps and missing data, a partial deletion strategy was applied with a 95% site coverage threshold. Branch support was evaluated through 1,000 bootstrap replicates.

### Specimen management and ethics statement

The specimen’s collection and study were conducted in strict accordance with the regulations for research in protected areas in Thailand. The research was granted by the Department of National Parks, Wildlife and Plant Conservation (DNP) (TS*0907.4/20335)*, Thailand. All protocols and objectives were fully disclosed to the DNP as part of the permit application process. All collected specimens after research finished were returned and deposited as voucher specimens to the DNP as in compliance with the permit requirements. No vertebrate animals were used in this study, and collection methods were designed to minimize impact on the *N. smilesii* and the surrounding ecosystem. The experimental protocols were approved by the Animal Experiment Committee of the Kasetsart University (Approval No. ACKU68-SCI-022). Spiders were anesthetized on ice before preserving in 95% ethanol and transported to the laboratory for DNA extraction and morphological analysis.

## Results

**Fig 3:**
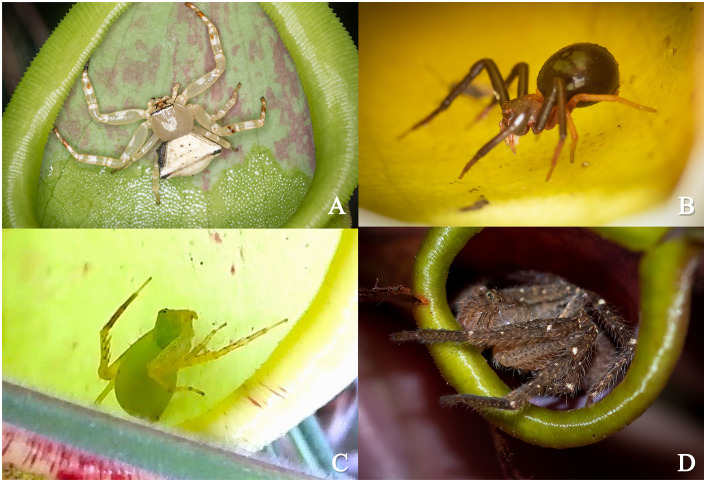
Spider species found inside the pitchers of *N. smilesii.* **(A)** *Thomisus* sp. (Pg020503), **(B)** *Henriksenia* sp. (Pg305312), **(C)** *Epidius* sp. (Pg071211), and **(D)** *Pseudopoda* sp. (Spa015909).

We identified three species of Thomisidae inhabiting the lower and upper regions of *N. smilesii* pitchers, exhibiting various colors and sizes. Two of these species closely resembled *Henriksenia nepenthicola* (28♀ 8♂) and *Thomisus nepenthiphilus* (5♀) (Figs 3A and 3B), although their specific identities could not be definitively confirmed. Only one individual of the third species was found and this species has not previously been reported to be associated with *Nepenthes*; its DNA sequences from the COI, 18S rRNA, and H3a genes were found to be closely related to *Epidius* sp. (1♀) (Fig 3C). Additionally, we identified a huntsman spider (N = 6, collected 1♀ 2♂) belonging to the *Pseudopoda* genus [31], marking the first reported instance of a Sparassidae in a relationship with *Nepenthes* (Fig 3D). The specimen codes for the identified species are as follows: Pg020503 (*Thomisus* sp.), Pg305312 (*Henriksenia* sp.), Pg071211 (*Epidius* sp.), and Spa015909 (*Pseudopoda* sp.) (Table in S3 Table).

### Behaviors

**Fig 4:**
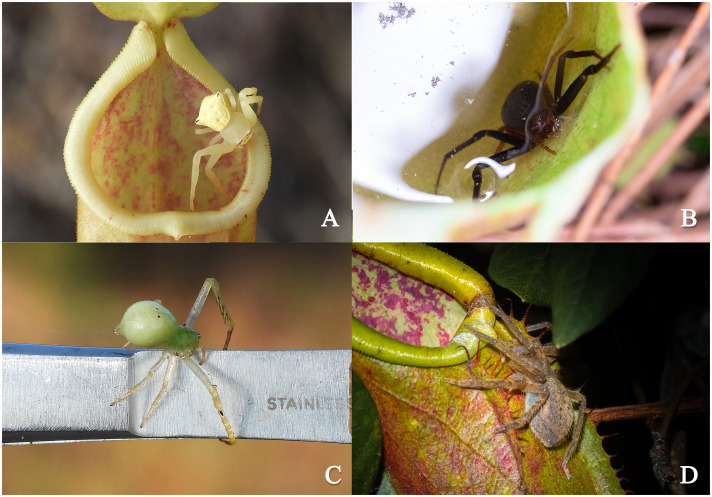
Behavior of four pitcher-associated spiders. **(A)** *Thomisus* sp. (Pg020503) waiting at the pitcher’s lid, **(B)** *Henriksenia* sp. (Pg305312) dived under the digestive fluid after being disrupted, **(C)** *Epidius* sp. (Pg071211) gently walked on the forceps without defensive, and **(D)** *Pseudopoda* sp. (Spa015909) came out at night.

Observations of four spider species associated with *N. smilesii* revealed distinct behavioral niches. While individuals were typically solitary per pitcher, multi-pitcher plants occasionally hosted multiple species. For example, the *Henriksenia* sp. and *Thomisus* sp. can be found at the same *N. smilesii* plant but different pitcher. Within the *Henriksenia* sp., we observed instances of cohabitation involving male-female pairs or females with offspring. The four species exhibited a clear gradient of *Nepenthes* association. *Henriksenia* sp. and *Thomisus* sp. showed the symbiotic behavior when disturbed by vibrations or forceps contact, both fled from their positions such as upper part or the pitcher’s lid (Fig 4A) into the pitcher’s bottom and submerged into digestive fluid (Fig. 4B). *Henriksenia* sp. responded significantly faster than *Thomisus* sp. (see video in S1 Video –S7 Video files). In contrast, *Pseudopoda* sp. and *Epidius* sp. lacked this diving reflex. *Epidius* sp. can inhabit the inner walls, but it showed no specialized defensive mechanisms or affinity for the digestive liquid (Fig 4C). In contrast, *Pseudopoda* sp. hung on the waxy wall perpendicular to the ground and would immediately jump off the pitcher when threatened. Nocturnal surveys indicated that *Pseudopoda* sp. utilizes the pitcher primarily as a diurnal shelter rather than a foraging site (Fig 4D).

### Whole mitochondrial genome

1. Mitogenome composition

**Fig 5:**
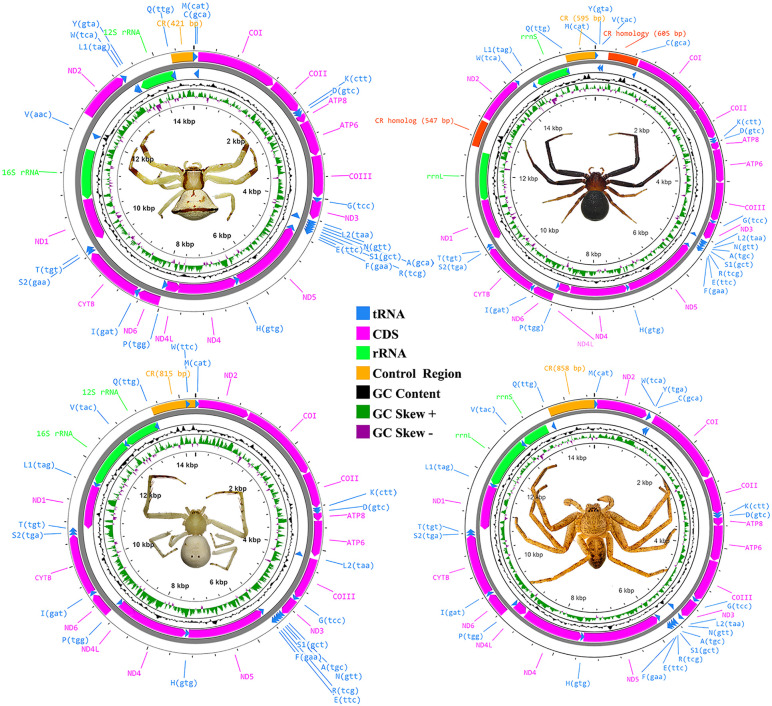
Complete mitogenome maps of four spiders associated with *Nepenthes.* Color-coding of PCGs, tRNA, rRNA genes, and the control region (CR) is indicated in the legend. GC content is shown with a black sliding window, while GC-skew is displayed with green and purple sliding windows. Anticodons of tRNAs are specified in parentheses in each figure.

**Fig 6:**
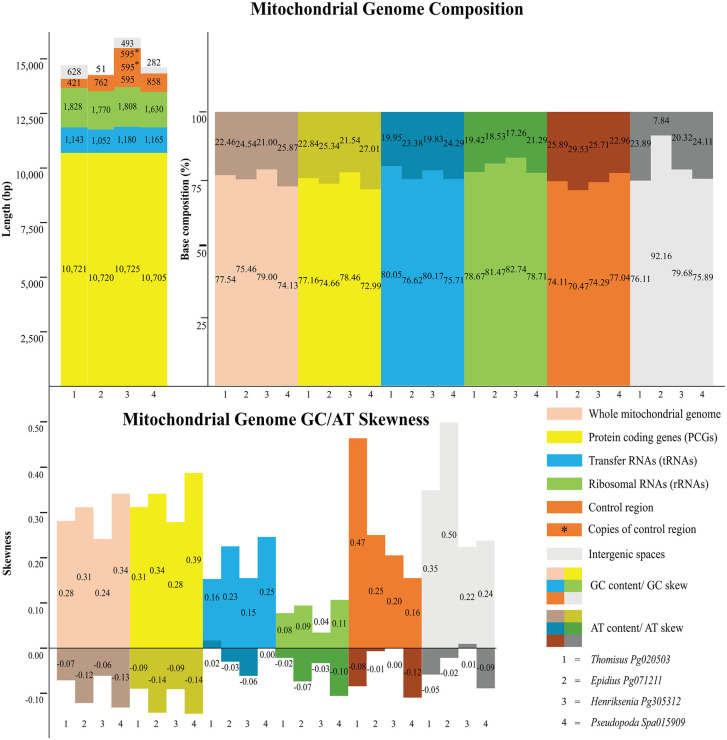
Overall mitochondrial genome compositions of four spiders associated with *Nepenthes.*

The complete mitogenome size of *Thomisus* sp. (PQ932624), *Epidius* sp. (PQ932623), *Henriksenia* sp. (PQ932622), and *Pseudopoda* sp. (PQ932621) were 14,731 bp, 14,289 bp, 15,888 bp, and 14,533 bp, respectively. These mitogenome sizes were typical compared to other spiders (Fig 5), e.g., *E. tricuspidata* (KU852748.1), *Oxytate striatipes* (KM507783.1), *T. onustus* (MW832852.1) and *Heriaeus mellotteei* (MW832850.1), which the genome size ranged between 14,407–14,530 bp. The mitogenomes were composed of 37 genes: 13 protein-coding genes (PCGs), two rRNA genes, and 22 tRNA genes. Twenty-two genes were located on the J-strand (major strand), while 15 genes were located on the N-strand (minor strand). The nucleotide composition of mitogenomes in the four spiders was similar to that of other arthropods, exhibiting an A + T bias (Table in S4 Table). All the four species exhibited a negative AT-skew and positive GC-skew, indicating a tendency toward T and G nucleotides (Fig 6). While the size of PCGs, tRNA and rRNA genes did not vary considerably among the four species of spiders, the size of the control region (CR) or D-loop varied across species, from 421 bp in *Thomisus* sp. to 858 bp in *Pseudopoda* sp. Both coding region (PGCs, tRNAs and rRNAs) and CR exhibited high A + T content (70.47–82.74%). PCGs, tRNAs and CR showed positive GC-skew while RNAs did not (Table in S4 Table and Fig 6).

1. Relative synonymous codon usage

**Fig 7:**
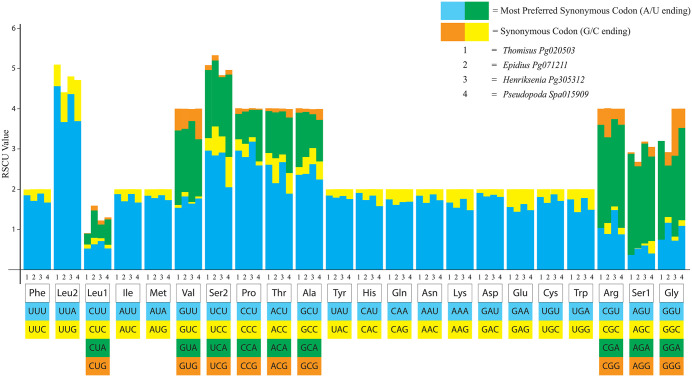
The relative synonymous codon usage of all four pitcher-dwelling spiders.

Codon usage in the PCGs of four spiders followed a pattern similar to that observed in other arachnids (Table 1). Start codons generally adhered to the ATN or TTN rule [16]. ATT was the most commonly used start codon, and TAA was the most frequently used stop codon, whereas TGA and TAG were rarely used. Incomplete stop codons, represented by a single T, were found in COI (*Pseudopoda* sp.), ND4 (*Epidius* sp. and *Pseudopoda* sp.), ND5, and CYTB (*Thomisus* sp. and *Henriksenia* sp.). The three most frequently used codons across all four spiders were UUA (Leu2), AUU (Ile), and UUU (Phe), which also corresponded to the three most frequently used amino acids. Analysis of the relative synonymous codon usage (RSCU) indicated a strong A + T bias over G + C. Codons GGG and GGC (Gly) were absent in *Thomisus* sp. (Fig 7).

**Table 1:** Gene order and features of the mitogenomes of four spider species associated with the *Nepenthes genus*. Size is in the units of bp, while minus sign indicates gene overlap.

Gene	Size (bp)					Intergenic space				Start/stop codon				
	*Thomisus*	*Epidius*		*Henriksenia*	*Pseudopoda*	*Thomisus*	*Epidius*	*Henriksenia*	*Pseudopoda*	*Thomisus*	*Epidius*	*Henriksenia*		*Pseudopoda*
Genome	14,731		14,289	15,888	14,533									
trnM(atg)	54		70	60	68	−29	0	−3	−4					
ND2	963		963	966	960	5	3	−2	11	ATT/TAA		ATT/TAG		ATG/TAA
trnW(tga)	56		53	51	76	6	112	186	−40					
trnY(tga)	54		*NA*	50	51	49	*NA*	−1	−10					
trnC(gca)	55		*NA*	59	59	0	*NA*	−5	−5					
COI	1,536		1,539	1,559	1,552	3	3	−35	22	TTA/TAA	ATA/TAA	TTA/TAG		TTA/T
COII	669		666	673	679	−2	8	−1	−15	TTG/TAA		TTG/TAG		
trnK(ctt)	60		56	57	53	−13	−18	−13	−9					
trnD(gac)	62		59	62	54	−5	−1	−5	−10					
ATP8	156		153	156	156	−1	−7	−7	−10	TTA/TAA			TTG/TAA	
ATP6	663		669	669	668	3	−21	4	12	ATA/TAA	ATG/TAA			
COIII	786		790	786	792	20	1	−1	−7	TTG/TAA				
trnG(gga)	66		48	59	55	0	10	−21	9					
ND3	357		336	351	350	−10	5	−18	−33	TTA/TAA	ATT/TAA	TTA/TAA		
trnL2(tta)	63		67	64	61	7	2	−1	−7					
trnN	44		56	71	59	−2	−24	−32	78					
trnA	45		57	64	56	−2	−8	−11	−5					
trnS1(agc)	54		62	53	51	−5	−8	−2	1					
trnR	52		60	70	56	−9	−5	−28	−19					
trnE(gaa)	54		52	61	53	−15	−15	−22	−4					
trnF	54		63	55	54	0	−12	−4	0					
ND5	1654		1651	1645	1642	−4	−2	−14	−7	ATT/T	ATA/T			ATT/T
trnH(cac)	57		52	56	60	−3	0	−5	−1					
ND4	1279		1276	1284	1288	−44	−101	4	12	TTG/T		TTG/TAA		ATA/T
ND4L	312		369	315	270	−5	1	−5	1	ATA/TAA	ATC/TAA	ATA/TAA		ATT/TAA
trnP(cca)	55		41	55	41	3	11	4	0					
ND6	432		435	432	432	−2	10	1	−2					
trnI(atc)	66		71	65	65	−16	8	17	14					
CYTB	1140		1137	1137	1140	−5	−1	−2	−8	ATA/T	TTA/TAG	ATG/T		ATT/TAA
trnS2(tca)	53		59	52	54	−2	0	−2	−2					
trnT(aca)	61		56	57	43	−12	−12	−10	−6					
ND1	921		918	924	921	0	−3	−7	−8	ATA/TAA	ATC/TAG	ATA/TAA		ATA/TAG
trnL1(cta)	56		48	46	57	−12	1	69	−1					
rrnL	1025		1029	1021	1055	278	0	706	−17					
trnV(gtt)	58		47	63	55	306	−3	686	7					
rrnS	721		722	724	691	−34	−39	−40	107					
trnQ(caa)	62		62	62	61	421	650	595	858					
CR	421		650	595	858									

1. Selection pressure revealed by nucleotide divergence in 13 PCGs of *Nepenthes* associated spiders and free-living spiders

**Fig 8:**
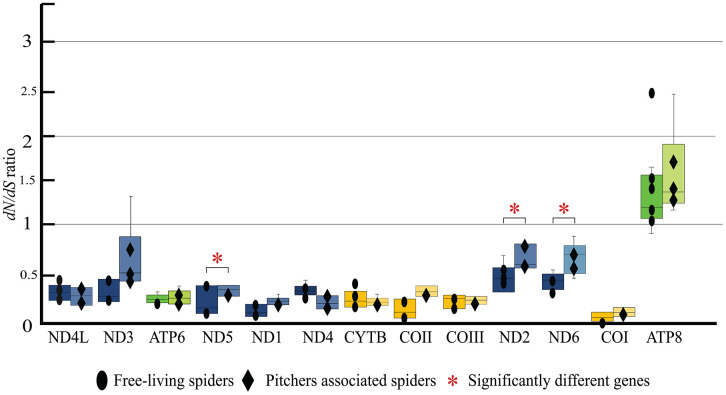
Comparison of *dN*/*dS* ratios for 13 mitochondrial PCGs between pitcher-associated and free-living spiders. Asterisks indicate significant differences (p < 0.05).

Analysis of *dN/dS* ratio (Fig 8 and Table in S5 Table) revealed strong purifying selection in 12 of the 13 PCGs, especially the genes in Complex III (CYTB), Complex IV (COI, COII, and COIII), and Complex V (ATP6). ATP8 is also the member of Complex V but it had exceptional high amino acids divergence (*dN*/*dS* > 1) indicating positive selection in this gene which was contrary to the others. However, the *dN*/*dS* of this gene was not significantly different between pitcher-associated and free-living spiders (*p =* 0.639). Three genes in the Complex I (ND genes), ND2, ND5, and ND6, were significantly differences (*p* = 0.031, *p* = 0.022, and *p* = 0.033) between pitcher-associated and free-living spiders.

1. Transfer RNA secondary structure

**Fig 9:**
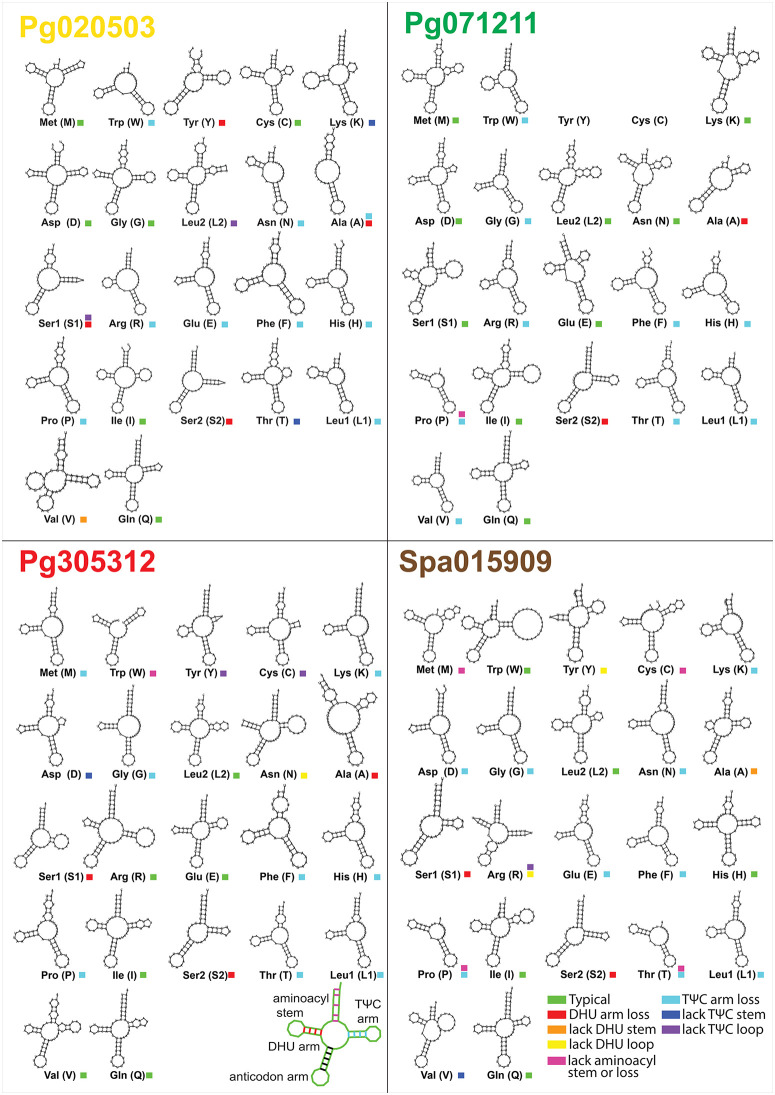
The analysis of the secondary structures of 22 tRNAs in the mitogenomes of the four spiders.

**Fig 10:**
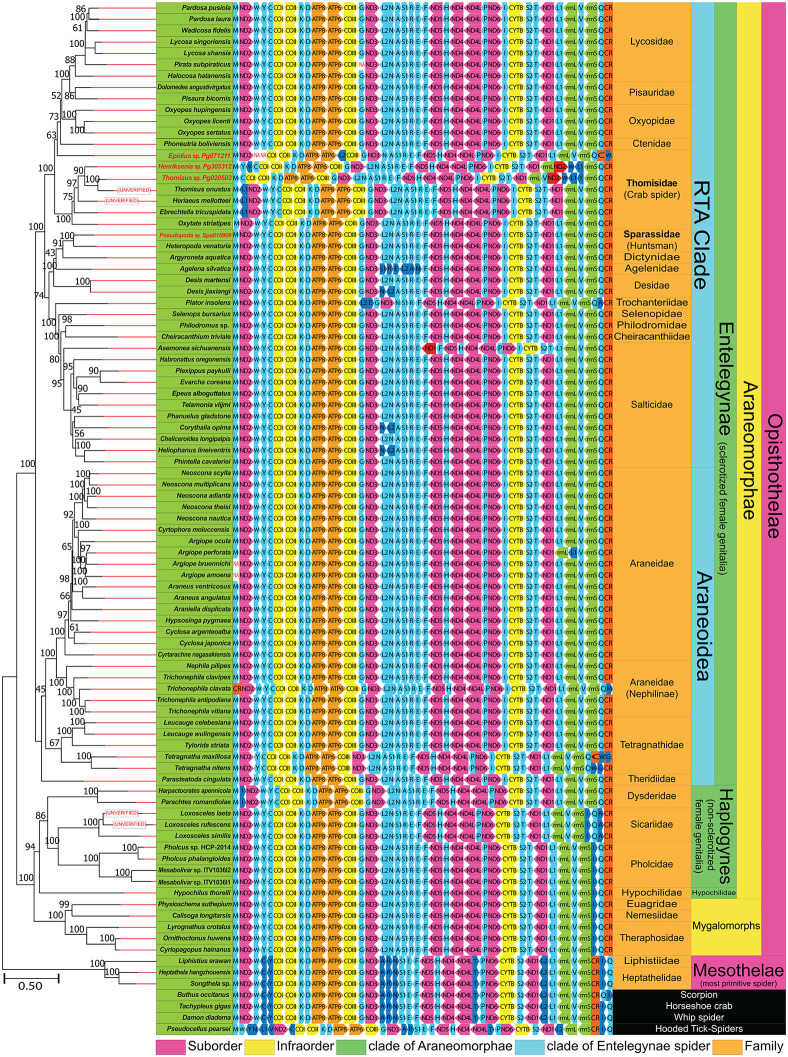
Maximum likelihood phylogenetic tree reconstructed from 13 protein-coding genes (PCGs), including gene arrangement maps for all analyzed spider and scorpion species. Light blue boxes represent tRNA genes.

The 22 tRNA genes of the four pitcher-associated spider mitogenomes were similar to those of other metazoan mitogenomes, except *Epidius* sp., which lacked trnY and trnC (encoding tyrosine and cysteine, respectively). Each tRNA corresponded to a specific amino acid, except for leucine and serine, which were each represented by two tRNAs (Fig 9). All the tRNAs were scattered throughout the mitogenome (Figs 10 and figure in S1 Fig). Analysis of the secondary structures of 22 tRNAs showed that only a minority of tRNAs were able to fold into typical cloverleaf structures. The number of tRNAs forming typical structures was as follows: five (22.7%) in *Pseudopoda* sp., six (27.3%) in *Thomisus* sp. and *Henriksenia* sp., and nine (41.0%) in *Epidius* sp. Only trnI and trnQ exhibited typical structures in all four spiders (Fig 9). On the other hand, a majority of tRNAs lack stems, loops, or entire arms – truncated tRNAs. Three types of such truncations were found: 1) Loss of the dihydrouracil (DHU) arm. 2) Loss of the TΨC (T stem-loop) or loss of the T loop. Only trnA of *Thomisus* sp. was completely armless. 3) Atypical aminoacyl stems, that were either reduced or complete loss of the aminoacyl stem (Fig 9).

1. Mitogenome rearrangement

The gene arrangement in *Pseudopoda* sp. was identical to those of other Entelegynae spiders. (Figs 10 and figure in S1 Fig). In contrast, *Thomisus* sp., *Epidius* sp., and *Henriksenia* sp. exhibited gene rearrangements. In *Thomisus* sp., there was a relocation of the ND2–trnW–trnY cluster and trnL1. In the *Henriksenia* sp., three sites were relocated: 1) ND2–trnW, 2) trnL1, and 3) trnV. In *Epidius* sp., the sequences expected to be trnY and trnC were missing and trnW was translocated to the D-loop region. As a result, ND2 was directly joined to COI with a 3-bp intergenic spacer, and trnQ was positioned inside the D-loop. Additionally, trnL2, which is typically located between ND3 and trnN, was moved to the region between ATP6 and COIII (Figs 10 and figure in S1 Fig).

1. Gene spacers and gene overlaps

**Fig 11:**
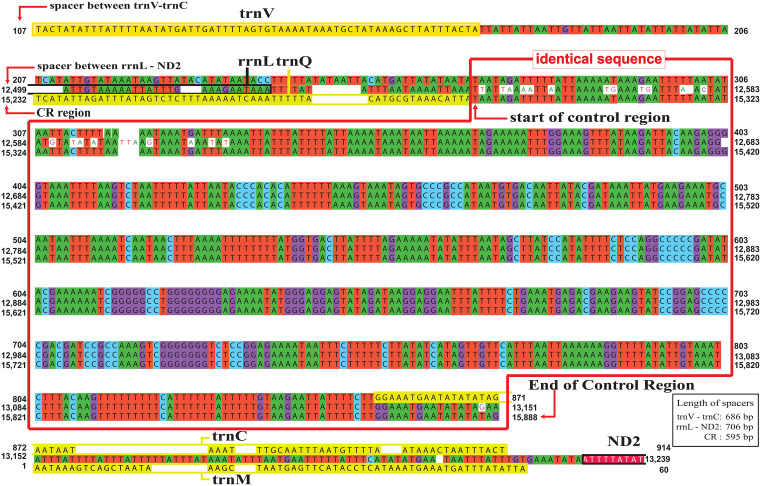
Comparison between the control region and its homologous sequences. Top sequence represents the spacer between trnV–trnC (595 bp, identical), middle sequence represents the spacer between rrnL–ND2 (595 bp, with 25 bp mismatches), while bottom sequence represents the control region.

*Henriksenia* sp. had nine intergenic spacer sites with a total length of 1,683 bp and contained the two longest spacers: 686 bp and 706 bp (figure in S1 Fig). Interestingly, both spacers exhibited high sequence similarity to a 595-bp region of the D-loop, spanning 577 bp and 595 bp (with 25 mismatches), respectively. (Fig 11). *Thomisus* sp. had 10 spacers with a total length of 644 bp, including two large spacers (278 bp and 306 bp). *Epidius* sp. had 12 spacers with a total length of 63 bp, and *Pseudopoda* sp. had 11 spacer sites with a total length of 274 bp, with the longest spacer (107 bp).

Gene overlaps are common in animal mitogenomes, likely due to selection pressure for genome compaction [16]. The number and total length of overlap sites varied among the studied species: *Thomisus* sp. had 22 sites (232 bp), *Epidius* sp. had 17 sites (280 bp), *Henriksenia* sp. had 27 sites (297 bp), and *Pseudopoda* sp. had 23 sites (230 bp).

1. Phylogenetic analysis based on PCGs and partial COI

**Fig 12:**
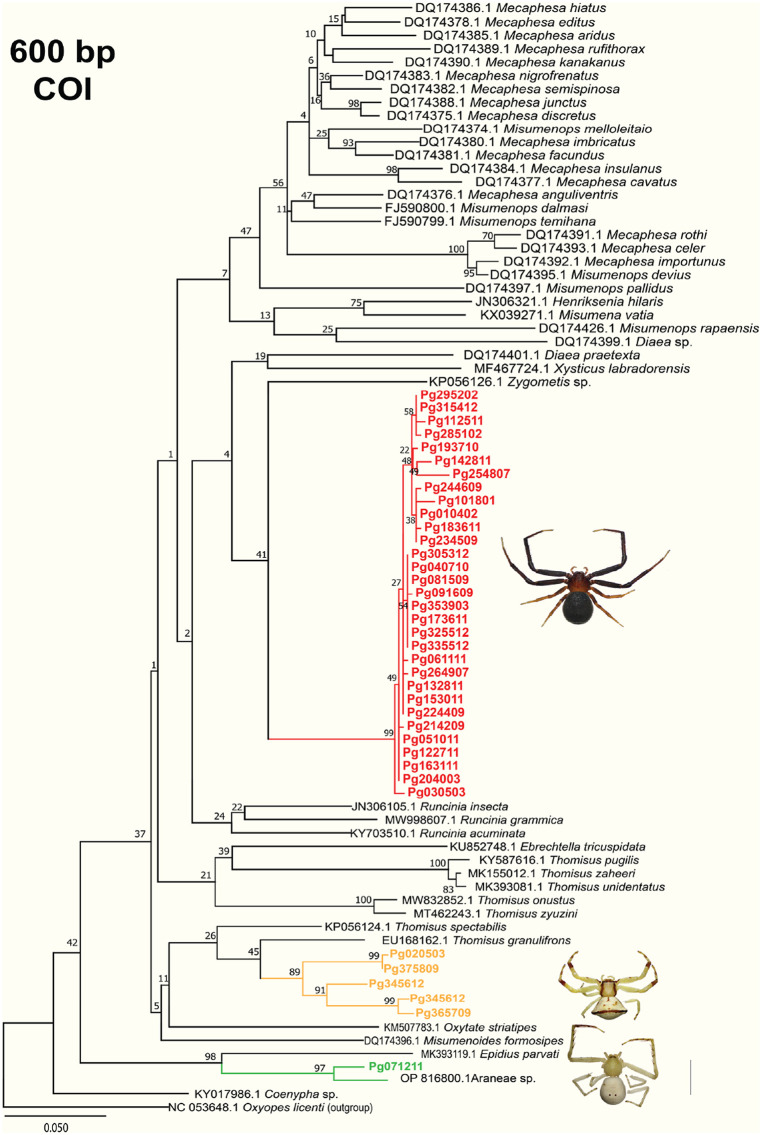
Maximum likelihood phylogenetic tree based on partial COI gene sequences with the Thomisidae samples available in GenBank.

A phylogenetic tree constructed using partial *COI* sequences of Thomisidae species available in GenBank (Fig 12) showed that all *Henriksenia* samples grouped together with low genetic distance (0.0–2.0%) (Table in S6 Table), indicating they belong to the same species. However, these samples did not cluster with the unverified *Henriksenia hilaris* (JN306321.1) from GenBank; instead, *H. hilaris* was more closely related to *Misumena vatia* (KX039271.1). Additionally, our *Henriksenia* (Pg305312) showed a 13% difference from *H. hilaris* (Table in S7 Table). Five *Thomisus* samples also grouped together with high bootstrap support (89–99%), but they exhibited a higher genetic distance (1.0–14%) compared to the *Henriksenia* samples. *Thomisus* sp. (Pg020503) was most closely related to *Thomisus granulifrons* (EU168162.1) with a 9% difference, indicating they are not the same species. Although *Epidius* sp. (Pg071211) grouped with *Epidius parvati* (MK393119.1), it was more closely related to an unidentified spider labeled “Araneae sp.” (OP816800.1). Overall, the COI phylogenetic tree clustered closely related species with high bootstrap support, while support was lower for distantly related samples. Unlike the tree based on 600 bp COI sequences, the tree constructed using all 13 PCGs from 83 mitochondrial genomes on NCBI exhibited strong bootstrap support, though fewer Thomisidae sequences were available for comparison. According to Fig 10, *Thomisus* sp., *Henriksenia* sp., and *T. onustus* were closely related and clustered within a monophyletic clade, while *Epidius* sp. was paraphyletic to the other Thomisidae species.

## Discussion

### Behavioral adaptations and ecological niches

The behavioral observation in this study revealed a distinct gradient of specialization among spiders associated with *N. smilesii*, ranging from casual inhabitants to highly adapted symbionts. This behavioral spectrum likely reflects varying degrees of co-evolutionary history with the host plant. The most striking behavioral adaptation observed was the diving behavior in *Henriksenia* sp. and *Thomisus* sp. This pitcher dwelling spider’s trait is a classic defensive mechanism for these spiders against predators, allowing them to exploit the digestive fluid for safety. Our finding that *Henriksenia* sp. responded faster than *Thomisus* sp. suggest a higher level of physiological and behavioral specialization. The cohabitant of *Henriksenia* and its mate or offspring in the same pitcher indicates that *N. smilesii* serves as a critical reproductive habitat in addition to foraging site [4,8]. Furthermore, the observation of multiple species inhabiting different pitchers on the same plant suggests niche partitioning, where interspecific competition is minimized by utilizing the plant’s structural complexity.

In contrast, the lack of diving reflex in *Epidius* sp. and *Pseudopoda* sp. classifies them as a generalized or facultative associate to the pitcher plant. *Epidius* sp. appears to utilize the inner wall for ambushing prey but lacks the diving behavior, suggesting its association is less dependent on the pitcher plants. The case of *Pseudopoda* sp. exhibited the ability to hang perpendicularly on the waxy wall highlights remarkable adhesion capabilities by a brush-like structure on tarsus [31]. Our nocturnal surveys observed suggest that this huntsman treats the pitcher as a diurnal shelter to avoid day-active predators or drought, rather than a foraging site. This represents a novel ecological role for huntsman spiders within *Nepenthes* communities, shifting the focus from “predator-prey” to “shelter-provider” dynamics.

### The mitochondrial genome characteristics of pitcher-associated spiders

The mitogenome size of the four spider specimens were comparable to those of other arthropods. Yet, the size of *Henriksenia* sp. (Pg305312) was notably larger than the others due to the addition of two control region-like sequences in intergenic spaces, making its genome size comparable to *Argyroneta aquatica* (NC026863.1) [32], which represents the largest recorded mitogenome (16,000 bp) in the Araneae order. The nucleotide composition indicated that the dominant bases were thymine (T) and guanine (G), a characteristic commonly observed in spiders of the suborder Opisthothelae and most of arthropods. The T-bias was driven by asymmetric mutational pressure in the mitochondrial DNA molecule. During the replication or transcription processes, one DNA strand (the template for the lagging strand) was exposed as single-stranded DNA for a longer duration than the other strand (leading strand), making the bases Adenine (A) and Cytosine (C) chemically less stable as they can be deaminated, and thus favoring the incorporation of Thymine (T) and Guanine (G) [33]. Furthermore, negative AT- skew was strongly associated with PCGs rather than RNA coding and non-coding regions. Interestingly, negative AT-skew preferentially located on the coding strand as most genes located on the heavy strand exhibited negative AT-skew while a few genes (ND1, ND4, ND4L and ND5) on the light strand exhibited positive AT-skew (Table in S4 Table). This finding is contrary to the report in primitive spiders from the Mesothelae suborder that have no or zero AT-skew and negative GC-skew [20]. Thus, the zero AT and positive GC-skew in primitive spiders potentially reflecting a different replication strategy to withstand the deamination process in the bases A and C in the primitive spiders compared to other spiders.

Concordant with other arthropods [33,34], the four spider genomes in this study exhibited high A + T content, which may stem from selection pressure associated with metabolic energy efficiency and transcriptional stability [33]. This high A + T content also associated with the use of ATG/ATT) and TTN (e.g., TTG) start codons and TAA biased stop codons. Some genes exhibited an incomplete stop codon, represented by a single T, which is commonly found in arthropod mitogenomes and is typically corrected during post-transcriptional modification [20,21]. The analysis of relative synonymous codon usage (RSCU) was also consistent with patterns reported in other arthropods, where synonymous codons preferentially utilize T or A bias in the third (wobble) position [20,21,35].

The tRNA secondary structure of the four spiders exhibited different patterns of truncated tRNAs, which have also been reported in other species from previous studies. Truncated tRNAs in metazoan mitogenomes are a common occurrence caused by selection pressure to minimize mitogenome size [36], and this is also observed in the Araneae order, where arm-less tRNAs have evolved and conserved for hundreds of millions of years, including in the suborder Mesothelae [36,37]. However, the evolutionary linkage of truncated tRNA patterns across species remains uncertain.

### The positive selection genes of the pitcher-associated spiders

To minimize confounding factors arising from broad evolutionary distances, we performed a comparative analysis of *dN*/*dS* ratios between pitcher-associated spiders (*n* = 4) and free-living spiders (*n* = 5) from the Thomisidae and Sparassidae families. Our findings align with previous mitochondrial studies on wolf spiders (*Lycosa* spp.), where 12 of the 13 protein-coding genes (PCGs) exhibited strong purifying selection (*dN*/*dS* < 1). Notably, genes in Complex IV (specifically COI, COII, and COIII) showed the lowest values, indicating high functional constraint. In contrast, ATP8—typically characterized by its short length and high mutational susceptibility—exhibited the most rapid evolution among the 13 PCGs (*dN*/*dS* > 1). However, this accelerated rate did not differ significantly between pitcher-associated and free-living spiders, suggesting a consistent evolutionary rate for ATP8 across these lineages. Conversely, NADH dehydrogenase subunits in Complex I (specifically ND5, ND2, and ND6) showed significantly accelerated evolutionary rates in pitcher-associated spiders compared to their free-living relatives. This suggests a lineage-specific molecular adaptation within the electron transport system, potentially driven by the unique physiological demands and specialized ecological niche of inhabiting carnivorous pitcher plants.

### Gene rearrangement and control region duplication in Entelegyne spiders

Unique gene rearrangements were identified in three Thomisidae species. Such rearrangements commonly occur in arachnids and have contributed significantly to the evolution of mitochondrial genome architecture across different spider lineages [18,19]. The occurrence of these rearrangements may be attributed to the Duplication-Random Loss (DRL) model, wherein errors during replication cause portions of the genome [38] to be duplicated and subsequently lost at random. This process causes previously adjacent genes, such as tRNAs or protein-coding genes (PCGs), to translocate or switch positions. Several unique cases of gene rearrangement have been reported within the Entelegynae clade: 1) tRNA shuffling and repositioning within the D-loop in *Tetragnatha maxillosa* (Tetragnathidae) [22,32], which is similar to the trnW translocation observed in *Epidius* sp.; 2) tRNA transposition in *Trichonephila clavata* [22] and *Argiope perforata*, or PCG transposition (e.g., ND1) in *Asemonea sichuanensis* (Fig. 10); 3) reverse transposition of tRNAs in *Corythalia opima* (Salticidae), *Heliophanus lineiventris*, *Desis jiaxiangi* (Desidae) [19], and *Plator insolens* (Trochanteriidae), or the formation of a large tRNA cluster into a new apomorphic gene boundary in *Agelena sylvatica* (Agelenidae); and 4) the loss of tRNA genes in *Pirata subpiraticus* [39], *Argiope bruennichi*, and *Argiope amoena* [40,41].

Spiders within the Thomisidae family exhibit diverse mitochondrial gene arrangements. For instance, a translocation of trnL1 to a position between trnW and trnY has been observed in three crab spiders—*E. tricuspidata*, *T. onustus*, and *H. mellotteei*—whereas *O. striatipes* retains the typical ancestral arrangement [27]. The gene transposition patterns in our three pitcher-associated spiders (*Thomisus*, *Henriksenia*, and *Epidius*) differ from these previously reported cases. Specifically, *Thomisus* (Pg020503) and *Henriksenia* (Pg305312) share a unique ND2*–*trnW gene block arrangement, while *Epidius* (Pg071211) exhibits a relocation of trnL2 to the region between ATP6 and COIII.

Furthermore, the duplication of two additional control regions (CRs) in *Henriksenia* represents an intriguing and rare phenomenon. While spider mitogenomes typically contain a single CR, certain species in the family Dysderidae (e.g., *Dysdera silvatica*, *Harpactocrates apennicola*, and *Parachtes romandiolae*) are known exceptions that possess an additional short CR sequence located between *trnL2* and *trnN* [32,42]. In contrast, *Henriksenia* (Pg305312) has duplicated the entire CR twice, a condition not previously reported in any other spider species. A similar duplication event has been observed in *Ixodes* ticks of the Australasian lineage [43], where two CRs were duplicated and subsequently evolved independently between species. This characteristic likely resulted from the Duplication-Random Loss (DRL) model via tandem duplication [44]. It has been suggested that multiple CRs may provide a selective advantage by allowing replication to initiate at several sites simultaneously, potentially increasing the mitogenome replication rate. If this hypothesis holds true, *Henriksenia* sp. and its paratype, which possess three CRs, may benefit from this accelerated replication capability.

### Evolutionary relationship of three pitcher-associated crab spiders

According to the phylogenetic tree based on 13 PCGs and gene block architecture, most crab spiders exhibit gene rearrangements, with the exception of *O. striatipes*. Our results show that *Epidius* sp. (Pg071211) is more genetically divergent than any other analyzed Thomisidae species, a finding corroborated by the *COI* phylogenetic analysis. Specifically, Pg071211 clustered with Araneae sp. (OP816800.1) and *Epidius parvati* [38] with high bootstrap support and a sequence distance of 2–12%, confirming its placement within the genus *Epidius*. The paraphyletic nature of *Epidius* sp. (Pg071211) may reflect its origin from a long-established lineage, suggesting that a formal re-classification at the subfamily level may be necessary to reflect these evolutionary distances.

On the other hand, sample Pg305312 and its paratype cannot be definitively identified as *Henriksenia* based solely on phylogenetic data, as they did not cluster with the reference *H. hilaris*. The current genus assignment for Pg305312 relies on external morphological similarities to *H. nepenthicola*, as there are currently no available taxonomic keys or molecular data for the genus *Henriksenia*. Similarly, the absence of DNA sequence data for *T. nepenthiphilus*—the only other *Thomisus* species known to inhabit pitcher plants—makes it impossible to determine its exact relationship to Pg020503. Our phylogenetic tree reveals that *Thomisus* is a highly diverse genus, with interspecific distances ranging from 1% to 18% (Table in S6 Table), while intraspecific distances remain notably low (Table in S7 Table). The taxonomy of Thomisidae remains challenging due to sparse and often outdated morphological data, leading to problematic species identification. Furthermore, discrepancies between published nomenclature and genetic databases are common [45]; for instance, many *Misumena* species shown in Fig. 12 were formerly classified as *Misumenops*. Consequently, more integrative studies combining detailed morphology with robust DNA sequencing are required to resolve the taxonomic ambiguities prevalent in these spider groups.

## Conclusions

This research provides the first comprehensive study of spiders associated with *Nepenthes smilesii* in Thailand. While *Henriksenia* sp. and *Thomisus* sp. demonstrated specialized symbiotic behavior, *Epidius* sp. and *Pseudopoda* sp. exhibited only limited association and lacked specialized diving adaptations. Notably, this study is the first to report that huntsman spiders (*Pseudopoda*) utilize pitcher plants as shelter, thereby expanding our understanding of ecological niches within *Nepenthes*-animal communities. Furthermore, the mitogenome sequences generated in this study help elucidate the evolutionary relationships within these complex spider taxonomic groups. Future research incorporating both mitogenomic data and behavioral observations will be essential to further understanding the co-evolution between spiders, pitcher plants, and other interacting organisms within these unique ecological niches.

## Supporting information

S1 Method Touchdown PCR for mitogenome amplification. (DOCX)

S1 Video Henriksenia lives inside the *N. smilesii* pitcher. (MP4)

S2 Video Henriksenia dives under the digestive fluid. (MP4)

S3 Video Thomisus live inside the *N. smilesii* pitcher. (MP4)

S4 Video Thomisus walking out from the *N. smilesii* pitcher. (MP4)

S5 Video Thomisus waiting at the pitcher lid. (MP4)

S6 Video Pseudopoda live inside the *N. smilesii* pitcher. (MP4)

S7 Video Pseudopoda running out from the *N. smilesii* pitcher. (MP4)

S8 Video Epidius live inside the *N. smilesii* pitcher. (MP4)

S1 Fig A comparison of mitogenome arrangements in seven Thomisidae and two Sparassidae species. (EPS)

S1 Table Primers used for completing the mitogenome of pitcher-dwelling spiders. C1-J-2123, Thom-F1, and Thom-F5 are adapted from [20] and [22], respectively.
(DOCX)

S2 Table A summary information of the 83 arachnid mitogenomes used in the analysis. (DOCX)

S3 Table Sample data of pitcher-associated spiders. (DOCX)

S4 Table Summary of the length, A + T content, and GC-skew for each genomic region in the mitogenomes of four pitcher-associated spider species: 1) *Thomisus* (Pg020503), 2) *Epidius* (Pg071211), 3) *Henriksenia* (Pg305312), and 4) *Pseudopoda* (Spa015909). (DOCX)

S5 Table *P*-values of the Wilcoxon Rank-Sum Test used to compare the differences between the dN/dS ratio of symbiotic vs. non-symbiotic spiders. (DOCX)

S6 Table Pairwise distances between the three pitcher-associated Thomisidae species based on partial COI gene sequences. (DOCX)

S7 Table Pairwise genetic distances between COI sequences of the analyzed Thomisidae species and other related arachnids. The distances range from 0–20%.
(DOCX)
