# Supplementary material for: Evolutionary adaptation and mitogenomic diversity of spiders associated with Nepenthes smilesii Pitcher Plants in Thailand
Source: PLoS One. 2026 May 4;21(5):e0348143. doi: 10.1371/journal.pone.0348143 (PMC13138635; doi:10.1371/journal.pone.0348143)
Supplement: S1 Method — (DOCX) [file pone.0348143.s001.docx]

**S1 Method. Touchdown PCR for mitogenome amplification**

The touchdown PCR program was used in this study, which begins with a high annealing temperature in the first cycle and decreases the annealing temperature by 1 °C per cycle until it is 10 °C lower than the starting temperature. This reduced temperature is maintained for the remaining cycles. This approach enhanced the primer specificity at optimal binding temperatures. The PCR protocol included: an initial denaturation at 95 °C for 3 minutes, followed by the touchdown protocol: cycle 1 with denaturation at 95 °C for 1 minute, annealing at the determined Tm of each primer pair for 30 seconds, and extension at 68 °C, for 1 minute per 1,000 bp of product size. Cycles 2–11 involved denaturation at 95 °C for 1 minute, decreasing annealing temperatures by 1 °C per cycle, and extension at 68 °C. Cycles 12–40 continued the denaturation, annealing at 10 °C below the initial Tm, and extension at 68 °C. A final extension was performed at 68 °C for 5 minutes, followed by cooling to 20 °C. An extension temperature of 68 °C, instead of the standard 72 °C, was used to reduce the depurination rate during amplification of long PCR products.
