# Supplementary material for: Evolutionary adaptation and mitogenomic diversity of spiders associated with Nepenthes smilesii Pitcher Plants in Thailand
Source: PLoS One. 2026 May 4;21(5):e0348143. doi: 10.1371/journal.pone.0348143 (PMC13138635; doi:10.1371/journal.pone.0348143)
Supplement: S1 Table — C1-J-2123, Thom-F1, and Thom-F5 are adapted from [20] and [22], respectively. (DOCX) [file pone.0348143.s011.docx]

**S1 Table.** Primers used for completing the mitogenome of pitcher-dwelling spiders. C1-J-2123, Thom-F1, and Thom-F5 are adapted from [20] and [22], respectively.

| Primer | Region | F/R sequence (5’ -3’) | Target size | Annealing temperature |
| --- | --- | --- | --- | --- |
| ThomF1-HCO | trnM–COI | AGGTCAGCTAATAAAGCTAATG  TAAACTTCAGGGTGACCAAAAAATCA | 2,000 | 48 |
| C1-J-2123-Thom R2.5 | COI–COII | GATCGAAATTTTAATACTTCTTTTTTTGA  GCATCHRCYTTYACYCCTAAA | 1,400 | 44 |
| Thom F2.5-Thom R2 | COII–COIII | TTTAGGRGTRAARGYDGATGC  GTTCTTTCCCGTACTACATCTC | 1,300 | 44 |
| Thom-F3-Thom-R3.5 | COIII–trnR | CGAGATGTAGTTCGGGAAAGAAC  TTAAGTCGAAATYAAGCCGC | 1,300 | 49 |
| Thom-F3.5-Thom-R3 | trnR–ND5 | GCGGCTTRATTTCGACTTAA  CAATTCCCTTTCTCAGCATGAC | 1,200 | 49 |
| Thom-F4-Thom-R4.5 | ND5–ND4 | GTCATGCTGAGAAAGGGAATTG  GAACGACTTCAAGCAAGAATTTAY | 1,540 | 49 |
| Thom-F4.5-Thom-R4 | ND4–ND6 | RTAAATTCTAGCTTGTAATCGTTC  CCATAATACAAACCTCGACC | 1,500 | 46 |
| ThomF5-Thom-R5 | trnI–rrnL | GTGCCTGAATAAAGGGTTAATTTG  CGATAAGACCCTATCGAACT | 2,600 | 49 |
| Thom-F6-Thom-R6.5 | rrnL–rrnS | CGGTCTGAACCCAATTCATG  GGTAAGTCGTAACATAGTTGATRTT | 1,000 | 47 |
| Thom-F6.5-RThom-F1 | rrnS–trnM | AAYATCAACTATGTTACGACTTACC  CATTAGCTTTATTAGCTGACHT | 1,600 | 45 |
| 18a2.0-18aR | 18sRNA | GATCCTTCCGCAGGTTCACCTAC  ATGGTTGCAAAGCTGAAAC | 650 | 50 |
| H3aF-H3aR | H3a | ATGGCTCGTACCAAGCAGACVGC  ATATCCTTRGGCATRATRGTGAC | 345 | 47 |
