# Supplementary material for: Evolutionary adaptation and mitogenomic diversity of spiders associated with Nepenthes smilesii Pitcher Plants in Thailand
Source: PLoS One. 2026 May 4;21(5):e0348143. doi: 10.1371/journal.pone.0348143 (PMC13138635; doi:10.1371/journal.pone.0348143)
Supplement: S2 Table — (DOCX) [file pone.0348143.s012.docx]

**S2 Table.** A summary information of the 83 arachnid mitogenomes used in the analysis

| **Suborder** | **Infraorder** | **Clade** | **Family** | **Species** | **Accession no.** | **Total length (bp)** | **%GC** |
| --- | --- | --- | --- | --- | --- | --- | --- |
| Mesothelae |  |  | Heptathelidae | *Songthela sp.* | MW822557.1 | 14,222 | 26 |
|  |  |  |  | *Heptathela hangzhouensis* | NC005924.1 | 14,215 | 28 |
|  |  |  | Liphistiidae | *Liphistius erawan* | JQ407803.1 | 14,197 | 32 |
| Opisthothelae | Mygalomorphs |  | Theraphosidae | *Cyriopagopus hainanus* | MN877932.1 | 13,874 | 30 |
|  |  |  |  | *Ornithoctonus huwena* | NC005925.1 | 13,874 | 30 |
|  |  |  |  | *Lyrognathus crotalus* | MN072398.1 | 13,886 | 31 |
|  |  |  | Nemesiidae | *Calisoga longitarsis* | EU523754.1 | 14,069 | 36 |
|  |  |  | Euagridae | *Phyxioschema suthepium* | NC020322.1 | 13,931 | 33 |
|  | Araneomorphae | Hypochilidae | Hypochilidae | *Hypochilus thorelli* | NC010777.1 | 13,990 | 30 |
|  |  | Haplogynes | Pholcidae | *Mesabolivar sp. ITV1036I1* | NC040859.1 | 14,941 | 29 |
|  |  |  |  | *Mesabolivar sp. ITV1036I2* | MH643813.1 | 14,845 | 32 |
|  |  |  |  | *Pholcus phalangioides* | NC020324.1 | 14,459 | 34 |
|  |  |  |  | *Pholcus sp. HCP-2014* | KJ782458.1 | 14,279 | 34 |
|  |  |  | Sicariidae | *Loxosceles similis* | NC042902.1 | 14,683 | 27 |
|  |  |  |  | *Loxosceles rufescens* | MK257773.1 | 15,210 | 36 |
|  |  |  |  | *Loxosceles laeta* | OP271740.1 | 14,925 | 34 |
|  |  |  | Dysderidae | *Parachtes romandiolae* | NC044099.1 | 14,220 | 29 |
|  |  |  |  | *Harpactocrates apennicola* | MN052924.1 | 14,213 | 28 |
|  |  | Entelegynae | Theridiidae | *Parasteatoda cingulata* | NC068059.1 | 14,260 | 21 |
|  |  | (Araneoidea) | Tetragnathidae | *Tetragnatha nitens* | NC028068.1 | 14,639 | 26 |
|  |  |  |  | *Tetragnatha maxillosa* | KP306789.1 | 14,578 | 25 |
|  |  |  |  | *Tylorida striata* | MN615900.1 | 14,422 | 28 |
|  |  |  |  | *Leucauge wulingensis* | NC061750.1 | 13,990 | 24 |
|  |  |  |  | *Leucauge celebesiana* | MN296353.1 | 13,901 | 23 |
|  |  |  | Araneidae | *Trichonephila vitiana* | MW178206.1 | 14,108 | 24 |
|  |  |  |  | *Trichonephila antipodiana* | MW178205.1 | 14,029 | 24 |
|  |  |  |  | *Trichonephila clavata* | AY452691.1 | 14,436 | 24 |
|  |  |  |  | *Trichonephila clavipes* | LC619787.1 | 14,902 | 23 |
|  |  |  |  | *Nephila pilipes* | MW178204.1 | 14,117 | 24 |
|  |  |  |  | *Cyrtarachne nagasakiensis* | NC028077.1 | 14,402 | 24 |
|  |  |  |  | *Cyclosa japonica* | MK512575.1 | 14,687 | 27 |
|  |  |  |  | *Cyclosa argenteoalba* | KP862583.1 | 14,575 | 26 |
|  |  |  |  | *Hypsosinga pygmaea* | NC028078.1 | 14,193 | 24 |
|  |  |  |  | *Araniella displicata* | NC064399.1 | 14,097 | 29 |
|  |  |  |  | *Araneus angulatus* | KU365988.1 | 14,205 | 25 |
|  |  |  |  | *Araneus ventricosus* | KM588668.1 | 14,617 | 27 |
|  |  |  |  | *Argiope amoena* | KJ607907.1 | 14,121 | 28 |
|  |  |  |  | *Argiope bruennichi* | NC024281.1 | 14,063 | 27 |
|  |  |  |  | *Argiope perforata* | NC044695.1 | 14,032 | 26 |
|  |  |  |  | *Argiope ocula* | MN331657.1 | 14,079 | 25 |
|  |  |  |  | *Cyrtophora moluccensis* | KM820884.1 | 14,344 | 26 |
|  |  |  |  | *Neoscona nautica* | NC029755.1 | 14,049 | 21 |
|  |  |  |  | *Neoscona theisi* | NC026290.1 | 14,156 | 25 |
|  |  |  |  | *Neoscona adianta* | NC029756.1 | 14,161 | 25 |
|  |  |  |  | *Neoscona multiplicans* | NC044653.1 | 14,074 | 25 |
|  |  |  |  | *Neoscona scylla* | MK086023.1 | 14,092 | 25 |
|  |  | Entelegynae | Salticidae | *Phintella cavaleriei* | MW540530.1 | 14,325 | 22 |
|  |  | (RTA) |  | *Heliophanus lineiventris* | MW832849.1 | 14,650 | 24 |
|  |  |  |  | *Cheliceroides longipalpis* | MH891570.1 | 14,334 | 21 |
|  |  |  |  | *Corythalia opima* | OQ281589.1 | 14,775 | 28 |
|  |  |  |  | *Phanuelus gladstone* | MT773150.1 | 14,458 | 25 |
|  |  |  |  | *Telamonia vlijmi* | KJ598073.1 | 14,601 | 23 |
|  |  |  |  | *Epeus alboguttatus* | NC042829.1 | 14,625 | 22 |
|  |  |  |  | *Evarcha coreana* | MK381265.1 | 14,333 | 24 |
|  |  |  |  | *Plexippus paykulli* | NC024877.1 | 14,316 | 27 |
|  |  |  |  | *Habronattus oregonensis* | NC005942.1 | 14,373 | 26 |
|  |  |  |  | *Asemonea sichuanensis* | NC061918.1 | 15,419 | 23 |
|  |  |  | Cheiracanthiidae | *Cheiracanthium triviale* | MN334527.1 | 14,595 | 22 |
|  |  |  | Philodromidae | *Philodromus sp.* | MZ507571.1 | 14,754 | 23 |
|  |  |  | Selenopidae | *Selenops bursarius* | NC024878.1 | 14,272 | 26 |
|  |  |  | Trochanteriidae | *Plator insolens* | OM397542.1 | 14,519 | 28 |
|  |  |  | Desidae | *Desis jiaxiangi* | NC060386.1 | 14,610 | 23 |
|  |  |  |  | *Desis martensi* | MW006608.1 | 14,662 | 23 |
|  |  |  | Agelenidae | *Agelena silvatica* | KX290739.1 | 14,776 | 26 |
|  |  |  | Dictynidae | *Argyroneta aquatica* | MK256613.1 | 16,000 | 28 |
|  |  |  | sparassidae | *Heteropoda venatoria* | OP650212.1 | 14,523 | 27 |
|  |  |  | Thomisidae | *Oxytate striatipes* | KM507783.1 | 14,407 | 22 |
|  |  |  |  | *Ebrechtella tricuspidata* | KU852748.1 | 14,530 | 24 |
|  |  |  |  | *Heriaeus mellotteei* | MW832850.1 | 14,502 | 24 |
|  |  |  |  | *Thomisus onustus* | MW832852.1 | 14,486 | 23 |
|  |  |  | Ctenidae | *Phoneutria boliviensis* | NC058921.1 | 14,724 | 28 |
|  |  |  | Oxyopidae | *Oxyopes sertatus* | NC025224.1 | 14,442 | 24 |
|  |  |  |  | *Oxyopes licenti* | NC053648.1 | 14,431 | 22 |
|  |  |  |  | *Oxyopes hupingensis* | NC046736.1 | 15,077 | 22 |
|  |  |  | Pisauridae | *Pisaura bicornis* | MN296112.1 | 15,281 | 22 |
|  |  |  |  | *Dolomedes angustivirgatus* | NC031355.1 | 14,783 | 23 |
|  |  |  | Lycosidae | *Halocosa hatanensis* | MT174468.2 | 14,257 | 22 |
|  |  |  |  | *Pirata subpiraticus* | KM486623.1 | 14,528 | 24 |
|  |  |  |  | *Lycosa shansia* | NC065747.1 | 14,638 | 21 |
|  |  |  |  | *Lycosa singoriensis* | NC065748.1 | 13,686 | 25 |
|  |  |  |  | *Wadicosa fidelis* | NC026123.1 | 14,741 | 24 |
|  |  |  |  | *Pardosa laura* | NC025223.1 | 14,513 | 23 |
|  |  |  |  | *Pardosa pusiola* | NC064110.1 | 14,284 | 24 |
