# Supplementary material for: Evolutionary adaptation and mitogenomic diversity of spiders associated with Nepenthes smilesii Pitcher Plants in Thailand
Source: PLoS One. 2026 May 4;21(5):e0348143. doi: 10.1371/journal.pone.0348143 (PMC13138635; doi:10.1371/journal.pone.0348143)
Supplement: S3 Table — (DOCX) [file pone.0348143.s013.docx]

**S3 Table.** Sample data of pitcher-associated spiders

| **Code** | **Organism** | **Collection Date** | **Sex** | **UTM(X)** | **UTM(Y)** | **Management** |
| --- | --- | --- | --- | --- | --- | --- |
| Pg010402 | *Henriksenia* | 22-Nov-21 | female | 798166.5 | 1869888 | DNA extraction, morphological measurement |
| Pg030503 | *Henriksenia* | 22-Nov-21 | male | 794769.1 | 1867837 | DNA extraction, morphological measurement, sex organ examines |
| Pg040710 | *Henriksenia* | 22-Nov-21 | male | 793775.5 | 1865966 | DNA extraction, morphological measurement, sex organ examines |
| Pg051011 | *Henriksenia* | 22-Nov-21 | female | 792207.2 | 1865792 | DNA extraction, morphological measurement |
| Pg061111 | *Henriksenia* | 22-Nov-21 | female | 792177.9 | 1865809 | DNA extraction, morphological measurement |
| Pg081509 | *Henriksenia* | 23-Nov-21 | female | 795967.4 | 1866600 | DNA extraction, morphological measurement, voucher specimen |
| Pg091609 | *Henriksenia* | 23-Nov-21 | female | 795967.9 | 1866602 | DNA extraction, morphological measurement |
| Pg101901 | *Henriksenia* | 23-Nov-21 | male | 799154.2 | 1867727 | DNA extraction, morphological measurement |
| Pg112511 | *Henriksenia* | 10-Jan-22 | male | 790773.1 | 1865998 | DNA extraction, morphological measurement |
| Pg122711 | *Henriksenia* | 10-Jan-22 | female | 790965.8 | 1865896 | DNA extraction, morphological measurement |
| Pg132811 | *Henriksenia* | 10-Jan-22 | female | 790958.1 | 1865889 | DNA extraction, morphological measurement |
| Pg142911 | *Henriksenia* | 10-Jan-22 | female | 791041.6 | 1865879 | DNA extraction, morphological measurement |
| Pg153011 | *Henriksenia* | 10-Jan-22 | female | 791113.6 | 1865864 | DNA extraction, morphological measurement, voucher specimen |
| Pg163111 | *Henriksenia* | 10-Jan-22 | female | 791397.7 | 1865860 | DNA extraction, morphological measurement |
| Pg173611 | *Henriksenia* | 10-Jan-22 | female | 792625.9 | 1865861 | DNA extraction, morphological measurement, sex organ examines |
| Pg183611 | *Henriksenia* | 10-Jan-22 | female | 792625.9 | 1865861 | DNA extraction, morphological measurement |
| Pg193710 | *Henriksenia* | 10-Jan-22 | male | 793030.2 | 1865896 | DNA extraction, morphological measurement, sex organ examines |
| Pg204003 | *Henriksenia* | 11-Jan-22 | female | 794473 | 1867856 | DNA extraction, morphological measurement |
| Pg214209 | *Henriksenia* | 11-Jan-22 | female | 794795.6 | 1866246 | DNA extraction, morphological measurement |
| Pg224409 | *Henriksenia* | 11-Jan-22 | male | 795105.5 | 1866391 | DNA extraction, morphological measurement |
| Pg234509 | *Henriksenia* | 11-Jan-22 | female | 795140.8 | 1866403 | DNA extraction, morphological measurement |
| Pg244609 | *Henriksenia* | 11-Jan-22 | female | 795190.2 | 1866419 | DNA extraction, morphological measurement |
| Pg254807 | *Henriksenia* | 11-Jan-22 | female | 796854.9 | 1866715 | DNA extraction, morphological measurement |
| Pg264907 | *Henriksenia* | 11-Jan-22 | male | 796893.8 | 1866716 | DNA extraction, morphological measurement |
| Pg285102 | *Henriksenia* | 12-Jan-22 | female | 798176 | 1869883 | DNA extraction, morphological measurement, sex organ examines |
| Pg295202 | *Henriksenia* | 12-Jan-22 | female | 798056.2 | 1869969 | DNA extraction, morphological measurement |
| Pg305312 | *Henriksenia* | 12-Jan-22 | female | 798885.8 | 1867101 | morphological measurement, Mitochondrial genome construction |
| Pg315412 | *Henriksenia* | 12-Jan-22 | female | 798788.3 | 1867078 | DNA extraction, morphological measurement |
| Pg325512 | *Henriksenia* | 10-Dec-22 | female | 798748.4 | 1867090 | DNA extraction, morphological measurement |
| Pg335512 | *Henriksenia* | 10-Dec-22 | male | 798748.4 | 1867090 | DNA extraction, morphological measurement, voucher specimen |
| Pg353903 | *Henriksenia* | 10-Dec-22 | female | 794473.2 | 1867856 | DNA extraction, morphological measurement |
| Spa015909 | *Pseudopoda* | 11-Dec-22 | male | 794769.1 | 1867837 | DNA extraction, morphological measurement |
| Spa026014 | *Pseudopoda* | 14-Apr-23 | male | 798199.8 | 1869869 | DNA extraction, morphological measurement |
| Spa036110 | *Pseudopoda* | 14-Apr-23 | female | 798734.8 | 1867092 | DNA extraction, morphological measurement, sex organ examines |
| Pg020503 | *Thomisus* | 22-Nov-21 | female | 792564.7 | 1865836 | morphological measurement, Mitochondrial genome construction |
| Pg275002 | *Thomisus* | 12-Jan-22 | female | 792464.1 | 1865831 | DNA extraction, morphological measurement |
| Pg345612 | *Thomisus* | 10-Dec-22 | female | 792162.2 | 1865810 | DNA extraction, morphological measurement |
| Pg365709 | *Thomisus* | 11-Dec-22 | female | 792289.5 | 1865858 | DNA extraction, morphological measurement |
| Pg375809 | *Thomisus* | 11-Dec-22 | female | 792626.7 | 1866955 | DNA extraction, morphological measurement, sex organ examines |
| Pg071211 | *Epidius* | 22-Nov-21 | female | 793503.5 | 1865903 | morphological measurement, Mitochondrial genome construction, sex organ examines |
