# Supplementary material for: Evolutionary adaptation and mitogenomic diversity of spiders associated with Nepenthes smilesii Pitcher Plants in Thailand
Source: PLoS One. 2026 May 4;21(5):e0348143. doi: 10.1371/journal.pone.0348143 (PMC13138635; doi:10.1371/journal.pone.0348143)
Supplement: S4 Table — (DOCX) [file pone.0348143.s014.docx]

**S4 Table.** Summary of the length, A+T content, and GC-skew for each genomic region in the mitogenomes of four pitcher-associated spider species: 1) *Thomisus* (Pg020503), 2) *Epidius* (Pg071211), 3) *Henriksenia* (Pg305312), and 4) *Pseudopoda* (Spa015909).

| **Region** | **A+T%** | | | | **G+C%** | | | | **GC-skew** | | | | **AT-skew** | | | |
| --- | --- | --- | --- | --- | --- | --- | --- | --- | --- | --- | --- | --- | --- | --- | --- | --- |
|  | ***1*** | ***2*** | ***3*** | ***4*** | ***1*** | ***2*** | ***3*** | ***4*** | ***1*** | ***2*** | ***3*** | ***4*** | ***1*** | ***2*** | ***3*** | ***4*** |
| Genome | 77.54 | 75.46 | 79.00 | 74.13 | 22.46 | 24.54 | 21.00 | 25.87 | 0.24 | 0.31 | 0.24 | 0.34 | -0.07 | -0.12 | -0.06 | -0.13 |
| PCGs | 77.16 | 74.66 | 78.46 | 72.99 | 22.84 | 25.34 | 21.54 | 27.01 | 0.31 | 0.34 | 0.28 | 0.39 | -0.09 | -0.14 | -0.09 | -0.14 |
| ND2 | 79.79 | 80.53 | 81.75 | 75.13 | 20.21 | 19.47 | 18.25 | 24.87 | 0.55 | 0.53 | 0.48 | 0.60 | -0.17 | -0.20 | -0.19 | -0.22 |
| COI | 70.83 | 69.78 | 71.31 | 69.11 | 29.17 | 30.22 | 28.69 | 30.89 | 0.22 | 0.21 | 0.22 | 0.28 | -0.24 | -0.26 | -0.25 | -0.29 |
| COII | 73.84 | 72.82 | 75.68 | 71.58 | 26.16 | 27.18 | 24.32 | 28.42 | 0.39 | 0.27 | 0.28 | 0.40 | -0.18 | -0.16 | -0.17 | -0.16 |
| ATP8 | 85.71 | 88.89 | 90.48 | 78.21 | 14.29 | 11.11 | 9.52 | 21.79 | 0.52 | 0.63 | 0.57 | 0.47 | -0.11 | -0.06 | -0.16 | -0.15 |
| ATP6 | 77.98 | 75.34 | 79.67 | 75.30 | 22.02 | 24.66 | 20.33 | 24.70 | 0.32 | 0.20 | 0.24 | 0.26 | -0.20 | -0.25 | -0.15 | -0.22 |
| COIII | 72.26 | 70.74 | 74.94 | 69.59 | 27.74 | 29.26 | 25.06 | 30.41 | 0.35 | 0.34 | 0.29 | 0.36 | -0.22 | -0.31 | -0.24 | -0.24 |
| ND3 | 81.52 | 77.68 | 83.63 | 74.57 | 18.48 | 22.32 | 16.37 | 23.14 | 0.44 | 0.47 | 0.43 | 0.38 | -0.27 | -0.46 | -0.23 | -0.24 |
| ND5 | 78.34 | 75.59 | 80.31 | 72.35 | 21.66 | 24.41 | 19.69 | 27.65 | 0.33 | 0.42 | 0.28 | 0.42 | 0.07 | 0.03 | 0.07 | -0.02 |
| ND4 | 80.38 | 74.84 | 79.75 | 44.78 | 19.62 | 25.16 | 20.25 | 55.22 | 0.30 | 0.43 | 0.38 | -0.33 | 0.10 | 0.03 | 0.09 | 0.71 |
| ND4L | 81.73 | 76.69 | 82.86 | 46.67 | 18.27 | 23.31 | 17.14 | 53.33 | 0.33 | 0.51 | 0.26 | -0.25 | 0.11 | 0.09 | 0.08 | 0.67 |
| ND6 | 82.87 | 81.15 | 83.80 | 76.85 | 17.13 | 18.85 | 16.20 | 23.15 | 0.51 | 0.54 | 0.51 | 0.68 | -0.12 | -0.20 | -0.17 | -0.17 |
| CYTB | 76.17 | 72.45 | 77.45 | 73.15 | 23.83 | 27.55 | 22.55 | 26.85 | 0.22 | 0.29 | 0.19 | 0.32 | -0.25 | -0.34 | -0.24 | -0.30 |
| ND1 | 78.94 | 75.60 | 80.30 | 73.94 | 21.06 | 24.40 | 19.70 | 26.06 | 0.11 | 0.27 | 0.09 | 0.26 | 0.15 | 0.09 | 0.18 | 0.03 |
| tRNAs | 80.05 | 76.62 | 80.17 | 75.71 | 19.95 | 23.38 | 19.83 | 24.29 | 0.16 | 0.23 | 0.15 | 0.25 | 0.02 | -0.03 | 0.00 | -0.06 |
| M | 72.22 | 77.14 | 76.67 | 73.53 | 27.78 | 22.86 | 23.33 | 26.47 | -0.07 | -0.13 | 0.00 | -0.22 | 0.13 | -0.07 | 0.17 | 0.00 |
| W | 84.00 | 79.25 | 80.39 | 75.00 | 16.00 | 20.75 | 17.65 | 25.00 | 0.00 | 0.45 | 0.11 | 0.16 | 0.10 | 0.05 | 0.22 | 0.02 |
| Y | 74.07 |  | 86.00 | 76.47 | 25.93 |  | 14.00 | 23.53 | 0.00 |  | -0.43 | 0.17 | 0.05 |  | -0.07 | -0.08 |
| C | 85.45 |  | 88.14 | 86.44 | 14.55 |  | 11.86 | 13.56 | -0.25 |  | 0.14 | 0.25 | -0.02 |  | 0.08 | -0.18 |
| K | 73.33 | 71.43 | 75.44 | 71.70 | 26.67 | 28.57 | 24.56 | 28.30 | 0.50 | 0.38 | 0.29 | 0.47 | -0.14 | -0.05 | 0.02 | -0.11 |
| D | 83.87 | 79.66 | 83.87 | 83.33 | 16.13 | 20.34 | 16.13 | 16.67 | 0.20 | 0.33 | 0.20 | 0.33 | 0.04 | -0.02 | 0.00 | 0.02 |
| G | 86.36 | 81.25 | 84.75 | 80.00 | 13.64 | 18.75 | 15.25 | 20.00 | 0.11 | 0.11 | 0.33 | 0.45 | 0.02 | 0.08 | -0.08 | 0.05 |
| L2 | 58.73 | 74.63 | 75.00 | 70.49 | 17.46 | 25.37 | 25.00 | 29.51 | -0.09 | 0.06 | 0.13 | 0.00 | -0.03 | -0.04 | -0.04 | -0.07 |
| N | 81.82 | 75.00 | 80.28 | 72.88 | 18.18 | 25.00 | 19.72 | 27.12 | 0.50 | 0.71 | 0.57 | 0.63 | 0.00 | 0.00 | 0.05 | 0.21 |
| A | 80.00 | 71.93 | 79.69 | 76.79 | 20.00 | 28.07 | 20.31 | 23.21 | 0.33 | 0.50 | 0.54 | 0.38 | 0.00 | 0.02 | 0.14 | -0.07 |
| S1 | 72.22 | 77.42 | 79.25 | 68.63 | 27.78 | 22.58 | 20.75 | 31.37 | 0.20 | 0.00 | 0.09 | 0.25 | 0.08 | 0.00 | 0.00 | 0.03 |
| R | 65.38 | 61.67 | 70.00 | 60.71 | 34.62 | 28.33 | 30.00 | 39.29 | 0.44 | 0.29 | 0.24 | 0.45 | 0.06 | 0.08 | 0.10 | -0.12 |
| E | 75.93 | 75.00 | 78.69 | 77.36 | 24.07 | 25.00 | 21.31 | 22.64 | 0.08 | 0.08 | 0.08 | 0.50 | -0.07 | -0.08 | -0.04 | -0.22 |
| F | 74.07 | 69.84 | 76.36 | 75.93 | 25.93 | 23.81 | 23.64 | 24.07 | 0.14 | -0.07 | 0.08 | -0.08 | -0.10 | -0.05 | -0.10 | -0.22 |
| H | 73.68 | 82.69 | 82.14 | 83.33 | 14.04 | 17.31 | 17.86 | 16.67 | -0.25 | 0.11 | 0.00 | 0.00 | 0.14 | -0.07 | 0.00 | 0.00 |
| P | 85.45 | 80.49 | 83.64 | 102.44 | 14.55 | 19.51 | 16.36 | 21.95 | 0.00 | 0.00 | 0.11 | -0.11 | -0.02 | -0.09 | -0.04 | -0.05 |
| I | 74.24 | 78.87 | 76.92 | 75.38 | 25.76 | 21.13 | 23.08 | 24.62 | 0.53 | 0.60 | 0.60 | 0.50 | 0.02 | 0.00 | -0.04 | 0.02 |
| S2 | 84.91 | 77.97 | 76.92 | 72.22 | 15.09 | 22.03 | 23.08 | 27.78 | 0.00 | 0.38 | 0.17 | 0.47 | 0.07 | -0.13 | -0.15 | -0.23 |
| T | 90.16 | 80.36 | 91.23 | 86.05 | 9.84 | 19.64 | 8.77 | 34.88 | 0.33 | 0.64 | 0.20 | 0.60 | 0.09 | 0.07 | 0.12 | -0.03 |
| L1 | 80.36 | 75.00 | 78.26 | 78.95 | 19.64 | 25.00 | 21.74 | 21.05 | -0.27 | 0.00 | -0.20 | 0.00 | 0.07 | -0.28 | -0.17 | -0.11 |
| V | 67.24 | 76.60 | 84.13 | 81.82 | 32.76 | 23.40 | 15.87 | 18.18 | -0.05 | 0.45 | 0.20 | 0.40 | -0.08 | 0.00 | -0.13 | -0.16 |
| Q | 80.65 | 79.03 | 80.65 | 73.77 | 19.35 | 20.97 | 19.35 | 26.23 | -0.33 | -0.08 | -0.17 | 0.00 | -0.04 | 0.06 | -0.04 | -0.02 |
| rRNAs | 78.67 | 81.47 | 82.74 | 78.71 | 19.42 | 18.53 | 17.26 | 21.29 | 0.08 | 0.09 | 0.04 | 0.11 | -0.02 | -0.07 | -0.03 | -0.10 |
| rrnL | 81.66 | 82.41 | 82.16 | 79.05 | 18.34 | 17.59 | 17.84 | 20.95 | 0.10 | 0.08 | 0.08 | 0.14 | 0.01 | -0.05 | -0.02 | -0.12 |
| rrnS | 78.38 | 80.16 | 83.58 | 78.09 | 21.62 | 19.84 | 16.42 | 21.91 | 0.03 | 0.09 | -0.03 | 0.05 | -0.06 | -0.11 | -0.05 | -0.06 |
| D-loop | 74.11 | 70.47 | 74.29 | 77.04 | 25.89 | 29.53 | 25.71 | 22.96 | 0.47 | 0.25 | 0.22 | 0.16 | -0.08 | -0.01 | 0.00 | -0.12 |
