# Supplementary material for: Evolutionary adaptation and mitogenomic diversity of spiders associated with Nepenthes smilesii Pitcher Plants in Thailand
Source: PLoS One. 2026 May 4;21(5):e0348143. doi: 10.1371/journal.pone.0348143 (PMC13138635; doi:10.1371/journal.pone.0348143)
Supplement: S5 Table — (DOCX) [file pone.0348143.s015.docx]

**S5 Table.** *P*-values of the Wilcoxon Rank-Sum Test used to compare the differences between the *dN/dS* ratio of symbiotic vs. non-symbiotic spiders*.*

| Gene | P_Value | Adjusted_P_Value | Significant |
| --- | --- | --- | --- |
| ND2 | 0.00472753 | 0.030728947 | TRUE |
| ND5 | 0.001692256 | 0.02199933 | TRUE |
| ND6 | 0.00762436 | 0.033038894 | TRUE |
| COI | 0.020071824 | 0.065233428 | FALSE |
| COII | 0.03982299 | 0.103539774 | FALSE |
| CYTB | 0.055711033 | 0.119858973 | FALSE |
| ND3 | 0.064539447 | 0.119858973 | FALSE |
| ND4L | 0.17881104 | 0.29056794 | FALSE |
| ATP8 | 0.442622048 | 0.639342958 | FALSE |
| ND1 | 0.741095292 | 0.9606338 | FALSE |
| ATP6 | 0.845031789 | 0.9606338 | FALSE |
| ND4 | 0.9606338 | 0.9606338 | FALSE |
| COIII | 0.9560797 | 0.9606338 | FALSE |
