# Supplementary material for: Evolutionary adaptation and mitogenomic diversity of spiders associated with Nepenthes smilesii Pitcher Plants in Thailand
Source: PLoS One. 2026 May 4;21(5):e0348143. doi: 10.1371/journal.pone.0348143 (PMC13138635; doi:10.1371/journal.pone.0348143)
Supplement: S6 Table — (DOCX) [file pone.0348143.s016.docx]

**S6 Table.** Pairwise distances between the three pitcher-associated Thomisidae species based on partial COI gene sequences.

| Code | Genus | 1 | 2 | 3 | 4 | 5 | 6 | 7 | 8 | 9 | 10 | 11 | 12 | 13 | 14 | 15 | 16 | 17 | 18 | 19 | 20 | 21 | 22 | 23 | 24 | 25 | 26 | 27 | 28 | 29 | 30 | 31 | 32 | 33 | 34 | 35 | 36 | 37 |
| --- | --- | --- | --- | --- | --- | --- | --- | --- | --- | --- | --- | --- | --- | --- | --- | --- | --- | --- | --- | --- | --- | --- | --- | --- | --- | --- | --- | --- | --- | --- | --- | --- | --- | --- | --- | --- | --- | --- |
| Pg020503 | *Thomisus* |  |  |  |  |  |  |  |  |  |  |  |  |  |  |  |  |  |  |  |  |  |  |  |  |  |  |  |  |  |  |  |  |  |  |  |  |  |
| Pg375809 |  | 0.01 |  |  |  |  |  |  |  |  |  |  |  |  |  |  |  |  |  |  |  |  |  |  |  |  |  |  |  |  |  |  |  |  |  |  |  |  |
| Pg275002 |  | 0.06 | 0.07 |  |  |  |  |  |  |  |  |  |  |  |  |  |  |  |  |  |  |  |  |  |  |  |  |  |  |  |  |  |  |  |  |  |  |  |
| Pg365709 |  | 0.07 | 0.07 | 0.01 |  |  |  |  |  |  |  |  |  |  |  |  |  |  |  |  |  |  |  |  |  |  |  |  |  |  |  |  |  |  |  |  |  |  |
| Pg345612 |  | 0.06 | 0.06 | 0.05 | 0.05 |  |  |  |  |  |  |  |  |  |  |  |  |  |  |  |  |  |  |  |  |  |  |  |  |  |  |  |  |  |  |  |  |  |
| Pg071211 | *Epidius* | 0.15 | 0.16 | 0.15 | 0.15 | 0.16 |  |  |  |  |  |  |  |  |  |  |  |  |  |  |  |  |  |  |  |  |  |  |  |  |  |  |  |  |  |  |  |  |
| Pg010402 | *Henriksenia* | 0.13 | 0.14 | 0.14 | 0.15 | 0.14 | 0.16 |  |  |  |  |  |  |  |  |  |  |  |  |  |  |  |  |  |  |  |  |  |  |  |  |  |  |  |  |  |  |  |
| Pg030503 |  | 0.15 | 0.15 | 0.16 | 0.16 | 0.15 | 0.18 | 0.02 |  |  |  |  |  |  |  |  |  |  |  |  |  |  |  |  |  |  |  |  |  |  |  |  |  |  |  |  |  |  |
| Pg040710 |  | 0.14 | 0.14 | 0.14 | 0.15 | 0.15 | 0.17 | 0.01 | 0.02 |  |  |  |  |  |  |  |  |  |  |  |  |  |  |  |  |  |  |  |  |  |  |  |  |  |  |  |  |  |
| Pg051011 |  | 0.14 | 0.14 | 0.15 | 0.15 | 0.15 | 0.17 | 0.01 | 0.02 | 0.01 |  |  |  |  |  |  |  |  |  |  |  |  |  |  |  |  |  |  |  |  |  |  |  |  |  |  |  |  |
| Pg061111 |  | 0.13 | 0.14 | 0.14 | 0.15 | 0.14 | 0.16 | 0.00 | 0.02 | 0.01 | 0.01 |  |  |  |  |  |  |  |  |  |  |  |  |  |  |  |  |  |  |  |  |  |  |  |  |  |  |  |
| Pg081509 |  | 0.13 | 0.14 | 0.14 | 0.15 | 0.14 | 0.16 | 0.01 | 0.02 | 0.01 | 0.01 | 0.01 |  |  |  |  |  |  |  |  |  |  |  |  |  |  |  |  |  |  |  |  |  |  |  |  |  |  |
| Pg091609 |  | 0.13 | 0.14 | 0.14 | 0.15 | 0.14 | 0.16 | 0.00 | 0.02 | 0.01 | 0.01 | 0.00 | 0.01 |  |  |  |  |  |  |  |  |  |  |  |  |  |  |  |  |  |  |  |  |  |  |  |  |  |
| Pg101801 |  | 0.14 | 0.14 | 0.14 | 0.15 | 0.14 | 0.16 | 0.00 | 0.02 | 0.01 | 0.01 | 0.01 | 0.01 | 0.01 |  |  |  |  |  |  |  |  |  |  |  |  |  |  |  |  |  |  |  |  |  |  |  |  |
| Pg112511 |  | 0.14 | 0.14 | 0.14 | 0.15 | 0.14 | 0.16 | 0.00 | 0.02 | 0.01 | 0.01 | 0.01 | 0.01 | 0.01 | 0.01 |  |  |  |  |  |  |  |  |  |  |  |  |  |  |  |  |  |  |  |  |  |  |  |
| Pg122711 |  | 0.14 | 0.15 | 0.14 | 0.15 | 0.14 | 0.16 | 0.01 | 0.02 | 0.01 | 0.01 | 0.01 | 0.01 | 0.01 | 0.01 | 0.01 |  |  |  |  |  |  |  |  |  |  |  |  |  |  |  |  |  |  |  |  |  |  |
| Pg132811 |  | 0.14 | 0.14 | 0.14 | 0.15 | 0.14 | 0.16 | 0.01 | 0.02 | 0.01 | 0.01 | 0.01 | 0.01 | 0.01 | 0.01 | 0.01 | 0.01 |  |  |  |  |  |  |  |  |  |  |  |  |  |  |  |  |  |  |  |  |  |
| Pg142811 |  | 0.13 | 0.14 | 0.14 | 0.15 | 0.14 | 0.16 | 0.00 | 0.02 | 0.01 | 0.01 | 0.00 | 0.01 | 0.01 | 0.01 | 0.01 | 0.01 | 0.01 |  |  |  |  |  |  |  |  |  |  |  |  |  |  |  |  |  |  |  |  |
| Pg153011 |  | 0.13 | 0.14 | 0.14 | 0.15 | 0.14 | 0.16 | 0.00 | 0.02 | 0.01 | 0.01 | 0.00 | 0.01 | 0.00 | 0.01 | 0.00 | 0.01 | 0.00 | 0.00 |  |  |  |  |  |  |  |  |  |  |  |  |  |  |  |  |  |  |  |
| Pg163111 |  | 0.13 | 0.14 | 0.14 | 0.15 | 0.14 | 0.16 | 0.00 | 0.01 | 0.01 | 0.01 | 0.00 | 0.01 | 0.00 | 0.01 | 0.00 | 0.01 | 0.01 | 0.01 | 0.00 |  |  |  |  |  |  |  |  |  |  |  |  |  |  |  |  |  |  |
| Pg173611 |  | 0.13 | 0.14 | 0.14 | 0.15 | 0.14 | 0.16 | 0.00 | 0.02 | 0.01 | 0.01 | 0.00 | 0.00 | 0.00 | 0.01 | 0.01 | 0.01 | 0.01 | 0.01 | 0.00 | 0.00 |  |  |  |  |  |  |  |  |  |  |  |  |  |  |  |  |  |
| Pg183611 |  | 0.13 | 0.14 | 0.14 | 0.15 | 0.14 | 0.16 | 0.00 | 0.02 | 0.01 | 0.01 | 0.00 | 0.01 | 0.00 | 0.00 | 0.00 | 0.01 | 0.01 | 0.00 | 0.00 | 0.00 | 0.00 |  |  |  |  |  |  |  |  |  |  |  |  |  |  |  |  |
| Pg193710 |  | 0.13 | 0.14 | 0.14 | 0.15 | 0.14 | 0.16 | 0.00 | 0.02 | 0.01 | 0.01 | 0.00 | 0.01 | 0.01 | 0.00 | 0.00 | 0.01 | 0.01 | 0.00 | 0.00 | 0.00 | 0.00 | 0.00 |  |  |  |  |  |  |  |  |  |  |  |  |  |  |  |
| Pg204003 |  | 0.13 | 0.14 | 0.14 | 0.15 | 0.14 | 0.16 | 0.01 | 0.02 | 0.01 | 0.01 | 0.01 | 0.01 | 0.01 | 0.01 | 0.01 | 0.01 | 0.01 | 0.01 | 0.01 | 0.00 | 0.01 | 0.01 | 0.01 |  |  |  |  |  |  |  |  |  |  |  |  |  |  |
| Pg214209 |  | 0.13 | 0.14 | 0.14 | 0.15 | 0.14 | 0.16 | 0.00 | 0.02 | 0.01 | 0.01 | 0.00 | 0.01 | 0.01 | 0.01 | 0.01 | 0.01 | 0.01 | 0.01 | 0.00 | 0.00 | 0.01 | 0.01 | 0.01 | 0.01 |  |  |  |  |  |  |  |  |  |  |  |  |  |
| Pg224409 |  | 0.13 | 0.14 | 0.14 | 0.15 | 0.14 | 0.16 | 0.00 | 0.02 | 0.01 | 0.01 | 0.00 | 0.01 | 0.00 | 0.01 | 0.00 | 0.01 | 0.00 | 0.00 | 0.00 | 0.00 | 0.00 | 0.00 | 0.00 | 0.01 | 0.00 |  |  |  |  |  |  |  |  |  |  |  |  |
| Pg234509 |  | 0.13 | 0.14 | 0.14 | 0.15 | 0.14 | 0.16 | 0.00 | 0.02 | 0.01 | 0.01 | 0.00 | 0.01 | 0.00 | 0.00 | 0.00 | 0.01 | 0.01 | 0.00 | 0.00 | 0.00 | 0.00 | 0.00 | 0.00 | 0.01 | 0.01 | 0.00 |  |  |  |  |  |  |  |  |  |  |  |
| Pg244609 |  | 0.13 | 0.14 | 0.14 | 0.15 | 0.14 | 0.16 | 0.00 | 0.02 | 0.01 | 0.01 | 0.00 | 0.01 | 0.01 | 0.00 | 0.01 | 0.01 | 0.01 | 0.00 | 0.00 | 0.00 | 0.00 | 0.00 | 0.00 | 0.01 | 0.01 | 0.00 | 0.00 |  |  |  |  |  |  |  |  |  |  |
| Pg254807 |  | 0.14 | 0.15 | 0.14 | 0.15 | 0.15 | 0.16 | 0.01 | 0.02 | 0.01 | 0.02 | 0.01 | 0.01 | 0.01 | 0.01 | 0.01 | 0.02 | 0.01 | 0.01 | 0.01 | 0.01 | 0.01 | 0.01 | 0.01 | 0.01 | 0.01 | 0.01 | 0.01 | 0.01 |  |  |  |  |  |  |  |  |  |
| Pg264907 |  | 0.14 | 0.14 | 0.15 | 0.16 | 0.14 | 0.17 | 0.01 | 0.02 | 0.01 | 0.01 | 0.01 | 0.01 | 0.01 | 0.01 | 0.01 | 0.02 | 0.01 | 0.01 | 0.01 | 0.01 | 0.01 | 0.01 | 0.01 | 0.01 | 0.01 | 0.01 | 0.01 | 0.01 | 0.02 |  |  |  |  |  |  |  |  |
| Pg285102 |  | 0.13 | 0.14 | 0.14 | 0.15 | 0.14 | 0.16 | 0.00 | 0.02 | 0.01 | 0.01 | 0.00 | 0.01 | 0.01 | 0.01 | 0.00 | 0.01 | 0.01 | 0.00 | 0.00 | 0.00 | 0.01 | 0.00 | 0.00 | 0.01 | 0.00 | 0.00 | 0.00 | 0.00 | 0.01 | 0.01 |  |  |  |  |  |  |  |
| Pg295202 |  | 0.13 | 0.14 | 0.14 | 0.15 | 0.14 | 0.16 | 0.00 | 0.02 | 0.01 | 0.01 | 0.00 | 0.01 | 0.01 | 0.01 | 0.00 | 0.01 | 0.01 | 0.00 | 0.00 | 0.00 | 0.01 | 0.00 | 0.00 | 0.01 | 0.00 | 0.00 | 0.00 | 0.00 | 0.01 | 0.01 | 0.00 |  |  |  |  |  |  |
| Pg305312 |  | 0.13 | 0.14 | 0.14 | 0.15 | 0.14 | 0.15 | 0.00 | 0.02 | 0.01 | 0.01 | 0.00 | 0.01 | 0.00 | 0.01 | 0.01 | 0.01 | 0.01 | 0.01 | 0.00 | 0.00 | 0.00 | 0.00 | 0.01 | 0.01 | 0.00 | 0.00 | 0.00 | 0.00 | 0.01 | 0.01 | 0.01 | 0.01 |  |  |  |  |  |
| Pg315412 |  | 0.13 | 0.14 | 0.14 | 0.15 | 0.14 | 0.16 | 0.00 | 0.02 | 0.01 | 0.01 | 0.00 | 0.01 | 0.01 | 0.01 | 0.00 | 0.01 | 0.01 | 0.00 | 0.00 | 0.00 | 0.01 | 0.00 | 0.00 | 0.01 | 0.00 | 0.00 | 0.00 | 0.00 | 0.01 | 0.01 | 0.00 | 0.00 | 0.01 |  |  |  |  |
| Pg325512 |  | 0.13 | 0.14 | 0.14 | 0.15 | 0.14 | 0.16 | 0.01 | 0.02 | 0.01 | 0.01 | 0.01 | 0.01 | 0.01 | 0.01 | 0.01 | 0.01 | 0.01 | 0.01 | 0.00 | 0.00 | 0.00 | 0.01 | 0.01 | 0.01 | 0.01 | 0.00 | 0.00 | 0.01 | 0.01 | 0.01 | 0.01 | 0.01 | 0.01 | 0.01 |  |  |  |
| Pg335512 |  | 0.13 | 0.14 | 0.14 | 0.15 | 0.14 | 0.16 | 0.01 | 0.02 | 0.01 | 0.01 | 0.01 | 0.01 | 0.01 | 0.01 | 0.01 | 0.01 | 0.01 | 0.01 | 0.01 | 0.01 | 0.01 | 0.01 | 0.01 | 0.01 | 0.01 | 0.01 | 0.01 | 0.01 | 0.01 | 0.01 | 0.01 | 0.01 | 0.01 | 0.01 | 0.01 |  |  |
| Pg353903 |  | 0.13 | 0.14 | 0.14 | 0.15 | 0.14 | 0.16 | 0.01 | 0.02 | 0.01 | 0.01 | 0.01 | 0.01 | 0.01 | 0.01 | 0.01 | 0.01 | 0.01 | 0.01 | 0.00 | 0.01 | 0.01 | 0.01 | 0.01 | 0.01 | 0.01 | 0.00 | 0.01 | 0.01 | 0.01 | 0.01 | 0.01 | 0.01 | 0.01 | 0.01 | 0.01 | 0.01 |  |
