# Supplementary material for: Evolutionary adaptation and mitogenomic diversity of spiders associated with Nepenthes smilesii Pitcher Plants in Thailand
Source: PLoS One. 2026 May 4;21(5):e0348143. doi: 10.1371/journal.pone.0348143 (PMC13138635; doi:10.1371/journal.pone.0348143)
Supplement: S7 Table — The distances range from 0–20%. (DOCX) [file pone.0348143.s017.docx]

**S7 Table.** Pairwise genetic distances between COI sequences of the analyzed Thomisidae species and other related arachnids. The distances range from 0–20%.

| Sample | 1 | 2 | 3 | 4 | 5 | 6 | 7 | 8 | 9 | 10 | 11 | 12 | 13 | 14 | 15 | 16 | 17 | 18 | 19 | 20 | 21 | 22 | 23 | 24 | 25 | 26 |
| --- | --- | --- | --- | --- | --- | --- | --- | --- | --- | --- | --- | --- | --- | --- | --- | --- | --- | --- | --- | --- | --- | --- | --- | --- | --- | --- |
| 1. *Thomisus onustus* |  |  |  |  |  |  |  |  |  |  |  |  |  |  |  |  |  |  |  |  |  |  |  |  |  |  |
| 2. *Thomisus zaheeri* | 0.13 |  |  |  |  |  |  |  |  |  |  |  |  |  |  |  |  |  |  |  |  |  |  |  |  |  |
| 3. *Thomisus unidentatus* | 0.13 | 0.01 |  |  |  |  |  |  |  |  |  |  |  |  |  |  |  |  |  |  |  |  |  |  |  |  |
| 4. *Thomisus spectabilis* | 0.11 | 0.13 | 0.14 |  |  |  |  |  |  |  |  |  |  |  |  |  |  |  |  |  |  |  |  |  |  |  |
| 5. *Thomisus pugilis* | 0.13 | 0.01 | 0.02 | 0.13 |  |  |  |  |  |  |  |  |  |  |  |  |  |  |  |  |  |  |  |  |  |  |
| 6. *Thomisus granulifrons* | 0.12 | 0.13 | 0.14 | 0.09 | 0.13 |  |  |  |  |  |  |  |  |  |  |  |  |  |  |  |  |  |  |  |  |  |
| 7. Pg020503 | 0.12 | 0.15 | 0.16 | 0.09 | 0.15 | 0.09 |  |  |  |  |  |  |  |  |  |  |  |  |  |  |  |  |  |  |  |  |
| 8. Pg375809 | 0.11 | 0.15 | 0.16 | 0.09 | 0.16 | 0.09 | 0.00 |  |  |  |  |  |  |  |  |  |  |  |  |  |  |  |  |  |  |  |
| 9. Pg275002 | 0.14 | 0.16 | 0.18 | 0.11 | 0.17 | 0.10 | 0.09 | 0.09 |  |  |  |  |  |  |  |  |  |  |  |  |  |  |  |  |  |  |
| 10. Pg345612 | 0.12 | 0.14 | 0.14 | 0.09 | 0.13 | 0.10 | 0.09 | 0.09 | 0.10 |  |  |  |  |  |  |  |  |  |  |  |  |  |  |  |  |  |
| 11. *Xysticus labradorensis* | 0.13 | 0.15 | 0.15 | 0.13 | 0.15 | 0.13 | 0.14 | 0.14 | 0.15 | 0.15 |  |  |  |  |  |  |  |  |  |  |  |  |  |  |  |  |
| 12. *Zygometis* sp. | 0.14 | 0.15 | 0.15 | 0.12 | 0.16 | 0.12 | 0.13 | 0.13 | 0.15 | 0.14 | 0.13 |  |  |  |  |  |  |  |  |  |  |  |  |  |  |  |
| 13. Pg061111 | 0.13 | 0.16 | 0.16 | 0.13 | 0.17 | 0.14 | 0.13 | 0.13 | 0.17 | 0.14 | 0.12 | 0.10 |  |  |  |  |  |  |  |  |  |  |  |  |  |  |
| 14. Pg254807 | 0.14 | 0.17 | 0.17 | 0.14 | 0.18 | 0.15 | 0.15 | 0.14 | 0.17 | 0.16 | 0.14 | 0.12 | 0.02 |  |  |  |  |  |  |  |  |  |  |  |  |  |
| 15. Pg285102 | 0.12 | 0.16 | 0.16 | 0.13 | 0.17 | 0.14 | 0.14 | 0.13 | 0.16 | 0.15 | 0.12 | 0.10 | 0.01 | 0.02 |  |  |  |  |  |  |  |  |  |  |  |  |
| 16. Pg305312 | 0.12 | 0.16 | 0.16 | 0.12 | 0.16 | 0.13 | 0.13 | 0.12 | 0.16 | 0.14 | 0.11 | 0.10 | 0.01 | 0.03 | 0.01 |  |  |  |  |  |  |  |  |  |  |  |
| 17. *Runcinia insecta* | 0.12 | 0.16 | 0.17 | 0.10 | 0.16 | 0.11 | 0.13 | 0.13 | 0.14 | 0.12 | 0.13 | 0.13 | 0.10 | 0.12 | 0.10 | 0.09 |  |  |  |  |  |  |  |  |  |  |
| 18*. Ebrechtella tricuspidata* | 0.12 | 0.13 | 0.14 | 0.12 | 0.13 | 0.15 | 0.14 | 0.15 | 0.17 | 0.14 | 0.18 | 0.13 | 0.14 | 0.15 | 0.14 | 0.13 | 0.13 |  |  |  |  |  |  |  |  |  |
| 19. *Mecaphesa kanakanus* | 0.11 | 0.13 | 0.14 | 0.11 | 0.14 | 0.13 | 0.13 | 0.13 | 0.15 | 0.12 | 0.15 | 0.12 | 0.13 | 0.15 | 0.13 | 0.12 | 0.12 | 0.13 |  |  |  |  |  |  |  |  |
| 20. *Misumenops melloleitaio* | 0.14 | 0.14 | 0.15 | 0.14 | 0.15 | 0.14 | 0.12 | 0.12 | 0.14 | 0.13 | 0.14 | 0.12 | 0.12 | 0.13 | 0.12 | 0.11 | 0.10 | 0.14 | 0.07 |  |  |  |  |  |  |  |
| 21. *Misumena vatia* | 0.13 | 0.14 | 0.16 | 0.17 | 0.15 | 0.14 | 0.16 | 0.16 | 0.16 | 0.14 | 0.14 | 0.12 | 0.14 | 0.16 | 0.14 | 0.13 | 0.13 | 0.15 | 0.12 | 0.11 |  |  |  |  |  |  |
| 22. *Henriksenia hilaris* | 0.14 | 0.17 | 0.17 | 0.15 | 0.17 | 0.14 | 0.16 | 0.15 | 0.17 | 0.14 | 0.15 | 0.13 | 0.13 | 0.15 | 0.13 | 0.13 | 0.13 | 0.15 | 0.12 | 0.11 | 0.08 |  |  |  |  |  |
| 23. *Epidius parvati* | 0.15 | 0.19 | 0.19 | 0.16 | 0.20 | 0.16 | 0.15 | 0.15 | 0.17 | 0.16 | 0.17 | 0.16 | 0.14 | 0.15 | 0.14 | 0.14 | 0.15 | 0.18 | 0.17 | 0.16 | 0.17 | 0.17 |  |  |  |  |
| 24. Araneae sp. | 0.14 | 0.16 | 0.17 | 0.15 | 0.17 | 0.15 | 0.14 | 0.14 | 0.17 | 0.16 | 0.16 | 0.14 | 0.16 | 0.17 | 0.16 | 0.15 | 0.15 | 0.16 | 0.14 | 0.15 | 0.15 | 0.17 | 0.12 |  |  |  |
| 25.Pg071211 | 0.14 | 0.16 | 0.17 | 0.15 | 0.17 | 0.15 | 0.14 | 0.14 | 0.16 | 0.15 | 0.15 | 0.13 | 0.15 | 0.16 | 0.15 | 0.14 | 0.14 | 0.15 | 0.14 | 0.14 | 0.15 | 0.16 | 0.11 | 0.02 |  |  |
| 26.*Oxytate striatipes* | 0.14 | 0.14 | 0.14 | 0.12 | 0.14 | 0.10 | 0.12 | 0.12 | 0.12 | 0.12 | 0.16 | 0.14 | 0.16 | 0.18 | 0.17 | 0.16 | 0.12 | 0.13 | 0.12 | 0.13 | 0.15 | 0.14 | 0.16 | 0.16 | 0.15 |  |
